# Neuro-fuzzy systems in internet of medical things: a systematic review on applications, taxonomy, challenges and open issues

**DOI:** 10.3389/fdgth.2026.1857965

**Published:** 2026-07-27

**Authors:** Richard Chilipa, Clement Nyirenda

**Affiliations:** 1 Department of Engineering, Malawi University of Science and Technology, Thyolo, Malawi; 2 Department of Computer Science, University of the Western Cape, Cape Town, South Africa

**Keywords:** clinical decision support, connected health, deployment-aware evaluation, edge-fog-cloud computing, evolving neuro-fuzzy systems, internet of medical things, neuro-fuzzy systems, soft computing

## Abstract

The growing demand for real-time, adaptive, and explainable analytics in the Internet of Medical Things (IoMT) has increased interest in neuro-fuzzy systems for connected healthcare. By combining neural learning with fuzzy inference, these systems can support predictive modelling while offering rule-based reasoning structures that require explicit evaluation for interpretability, stability, and clinical usability. This paper presents a systematic review based on structured database searches, eligibility screening, quality appraisal, and narrative synthesis of 55 peer-reviewed journal articles published between January 2020 and July 2025 on neuro-fuzzy systems in IoMT and connected-health contexts. The included articles were retrieved from MDPI, SpringerLink, ScienceDirect/Elsevier, IEEE Xplore, Wiley Online Library, and Taylor & Francis, with Google Scholar used only for verification and citation tracing. An article-level, deployment-aware taxonomy was developed to classify the evidence by architecture, application, design, deployment environment, and reported metrics. Neural-network-based optimization was the most frequently reported architecture, accounting for 28 articles (50.9%), followed by hybrid neuro-fuzzy systems with 11 articles (20.0%), deep neuro-fuzzy systems with 9 articles (16.4%), and evolving neuro-fuzzy systems with 7 articles (12.7%). This distribution indicates that the evidence base is still shaped mainly by static or offline neuro-fuzzy designs, while evolving architectures for streaming, non-stationary, and patient-specific IoMT data remain comparatively underexplored. Predictive systems formed the largest application category, followed by detection-oriented systems. Deployment reporting remained limited, with 34 of the 55 included articles (61.8%) not specifying an execution environment, while only 6 articles (10.9%) reported latency or processing-time evidence. Overall, the evidence remains mainly retrospective, experimental, simulation-based, benchmark-driven, or prototype-level. The review identifies recurring gaps in online adaptability, interoperability, deployment-aware performance evaluation, and practical clinical interpretability. Although neuro-fuzzy systems show promise as adaptive models for connected-health applications, the current evidence does not yet establish routine clinical readiness or clinical effectiveness. Future work should strengthen evolving neuro-fuzzy modelling, prospective validation, deployment-aware evaluation, safety assessment, interpretability assessment, and integration with electronic health record and telemedicine workflows.

## 1 Introduction

The healthcare sector is undergoing a substantial digital transformation, shifting from conventional models centred on episodic care and manual record keeping toward intelligent, connected, and data-driven systems that support continuous monitoring, timely intervention, and personalized care (1, 2). A key enabler of this transition is the Internet of Medical Things (IoMT), which comprises interconnected medical devices, wearable technologies, implantable sensors, mobile health platforms, and health information systems that acquire, transmit, and exchange physiological and behavioural data across patients, clinicians, and healthcare infrastructures (3, 4). Through such connectivity, IoMT supports remote patient monitoring, chronic disease management, telemedicine, early warning systems, and more responsive clinical decision making (5, 6).

The growth of IoMT reflects broader changes in healthcare service delivery. Connected medical devices are increasingly being used to extend care beyond hospitals into homes, community settings, ambulatory care, and remote-monitoring environments. Although market estimates vary, they consistently indicate rapid expansion: earlier estimates placed the IoMT market at approximately USD 158.1 billion by 2022, while more recent estimates valued the global IoMT market at USD 230.69 billion in 2024 and projected growth to USD 658.57 billion by 2030 (7, 8). This expansion is driven by rising demand for remote patient monitoring, wearable technologies, telehealth integration, cost-efficient care delivery, and the increasing need to manage chronic diseases outside traditional hospital settings. For health systems, the expected impact is not limited to more devices. IoMT is expected to support earlier detection of clinical deterioration, improved care continuity, more personalized disease management, reduced pressure on physical healthcare infrastructure, and wider access to services for underserved or hard-to-reach populations (2, 9). IoMT value depends on continuous processing, not only data collection. These systems generate heterogeneous, noisy, high-volume, and temporally dynamic data from devices with different sampling rates, protocols, battery limits, and reliability profiles. This creates challenges for conventional methods under uncertainty, nonlinearity, missing values, and non-stationary conditions (10). IoMT therefore needs intelligent models that support accurate prediction, adaptive learning, evaluated explainability, and deployment-aware operation across edge, fog, cloud, and hybrid healthcare infrastructures.

To address these challenges, artificial intelligence (AI) and machine learning (ML) have become integral to IoMT platforms, improving predictive performance, responsiveness, and adaptive decision support (3, 11). Among the earlier intelligent approaches, artificial neural networks (ANNs) gained prominence because of their strong pattern-recognition capabilities and their success in tasks such as medical image analysis, physiological signal interpretation, and disease classification (12, 13). In parallel, fuzzy logic systems provided an effective means of representing and reasoning with imprecise clinical knowledge through rule-based structures that can be inspected and tested for interpretability. By operating on linguistic variables such as “moderate fever” or “elevated blood pressure,” fuzzy systems can support human-readable *if-then* reasoning when rule bases remain concise, stable, and clinically meaningful in settings characterized by ambiguity and uncertainty (14, 15). Nevertheless, these paradigms also exhibit important limitations when applied independently. Neural networks are often criticized for limited transparency, while fuzzy systems may become difficult to scale in high-dimensional and data-intensive environments (16, 17).

Neuro-fuzzy systems (NFS) emerged as a hybrid approach that combines the learning capability of neural models with the rule-based reasoning structure of fuzzy inference (18). Such a combination is especially relevant to IoMT, where intelligent systems are expected not only to learn from noisy, nonlinear, and heterogeneous data streams, but also to support clinically meaningful and explainable reasoning. For this reason, NFS have been applied in diagnostic systems, patient monitoring, risk prediction, and treatment recommendation tasks, where predictive performance and evaluated interpretability are both important (19–21). Their relevance in IoMT, however, extends beyond model-level predictive capability. In practical deployments, IoMT systems must also operate under systems-level constraints such as low latency, scalability, interoperability, energy efficiency, privacy, security, and adaptability across distributed edge, fog, and cloud environments. Accordingly, the study of NFS in IoMT must be approached not only from an algorithmic perspective, but also from the perspective of deployment feasibility, clinical workflow integration, and long-term service delivery.

Despite increasing interest in the application of neuro-fuzzy systems within IoMT, the literature remains fragmented. Existing reviews have either focused on specific healthcare applications or examined intelligent IoMT systems more broadly without providing a dedicated and deployment-aware treatment of NFS. For example, (15) reviewed NFS in disease diagnosis, but placed primary emphasis on predictive performance rather than deployment-related issues such as latency, interoperability, and resource constraints. Similarly, (19) surveyed IoT-enabled fuzzy systems for healthcare, but neuro-fuzzy systems received only limited attention and no dedicated architectural or methodological taxonomy was established. The review in (21) examined fuzzy intelligence and edge computing in infectious disease monitoring, yet its scope was largely restricted to pandemic-oriented applications and did not provide a generalizable perspective on NFS design in IoMT. In (20), the emphasis was on explainable fuzzy inference for diagnosis, with limited discussion of distributed deployment, scalability, and execution constraints. Likewise, (22) presented a taxonomy of fuzzy deep learning architectures for uncertain medical data, but provided limited insight into standalone NFS models, their deployment across edge, fog, and cloud layers, or the associated trade-offs involving latency, energy consumption, and memory efficiency.

Taken together, these limitations highlight the absence of a unified, deployment-aware review framework for systematically understanding how NFS are designed, evaluated, and applied within IoMT ecosystems. In this review, *deployment-aware* refers to the explicit consideration of the conditions under which a neuro-fuzzy system would operate in realistic connected-healthcare environments, including latency, memory footprint, energy consumption, communication overhead, throughput, robustness to missing or noisy sensor data, interoperability, privacy, security, and integration with electronic health records, telemedicine, or remote-monitoring workflows. This perspective is important because the future impact of IoMT on healthcare service delivery will depend not only on predictive accuracy, but also on whether intelligent models can operate safely, efficiently, and transparently within real clinical and distributed computing environments. To address this gap, the present review provides a structured survey of NFS in IoMT by synthesizing the included peer-reviewed journal-article evidence across architectural paradigms, healthcare application domains, and deployment environments, while critically examining implementation challenges, evaluation practices, and open research issues. The main contributions of this review are as follows:

- a structured, article-level and deployment-aware taxonomy for organizing peer-reviewed journal articles on neuro-fuzzy systems in IoMT according to architectural paradigm, deployment environment, and healthcare application domain;
- a critical review framework for examining the implementation, evaluation, and deployment considerations reported across the included studies, with particular attention to model design, performance assessment, interpretability, interoperability, and clinical applicability; and
- a synthesis-oriented discussion of emerging knowledge gaps and future research directions for advancing adaptive, auditable, scalable, and deployment-aware neuro-fuzzy systems for connected healthcare service delivery.

The remainder of this paper is organized as follows. Section 2 introduces the Internet of Medical Things. Section 3 presents the fundamentals of neuro-fuzzy systems. Section 4 reviews related surveys and identifies the research gap. Section 5 describes the systematic review methodology. Section 6 presents the proposed taxonomy. Section 7 discusses the main findings. Section 8 presents the challenges, open issues, and future research directions. Finally, Section 9 concludes the paper.

## 2 Internet of medical things

### 2.1 Overview of IoMT

The Internet of Medical Things (IoMT) refers to a connected healthcare ecosystem of medical sensors, wearable and implantable devices, mobile platforms, clinical equipment, communication networks, and software services that acquire, transmit, process, and exchange health-related data (3). By combining sensing, connectivity, and computing, IoMT supports remote patient monitoring, chronic disease management, telemedicine, emergency response, and data-driven clinical decision support (4–6). In this review, IoMT is treated broadly to include related connected-health contexts such as smart healthcare, medical IoT, wearable health monitoring, wireless body sensor networks, and edge-, fog-, or cloud-enabled healthcare systems.

IoMT applications typically involve continuous or periodic acquisition of physiological and contextual data, followed by transmission to local gateways, edge or fog nodes, cloud platforms, or clinical information systems. These data may support early risk detection, patient-status tracking, personalized care, and timely clinical intervention (23, 24). Examples include wearable cardiac monitoring, glucose monitoring, fall detection, smart inhalers, insulin pumps, telemedicine platforms, and hospital-based connected equipment (25–27). However, IoMT systems also introduce important challenges, including data heterogeneity, device interoperability, latency, privacy, security, energy constraints, and the need for trustworthy clinical integration. These challenges motivate the use of intelligent models that can operate under uncertainty while remaining adaptable and explainable when their rule bases are evaluated properly.

### 2.2 IoMT architecture

**Figure 1:**
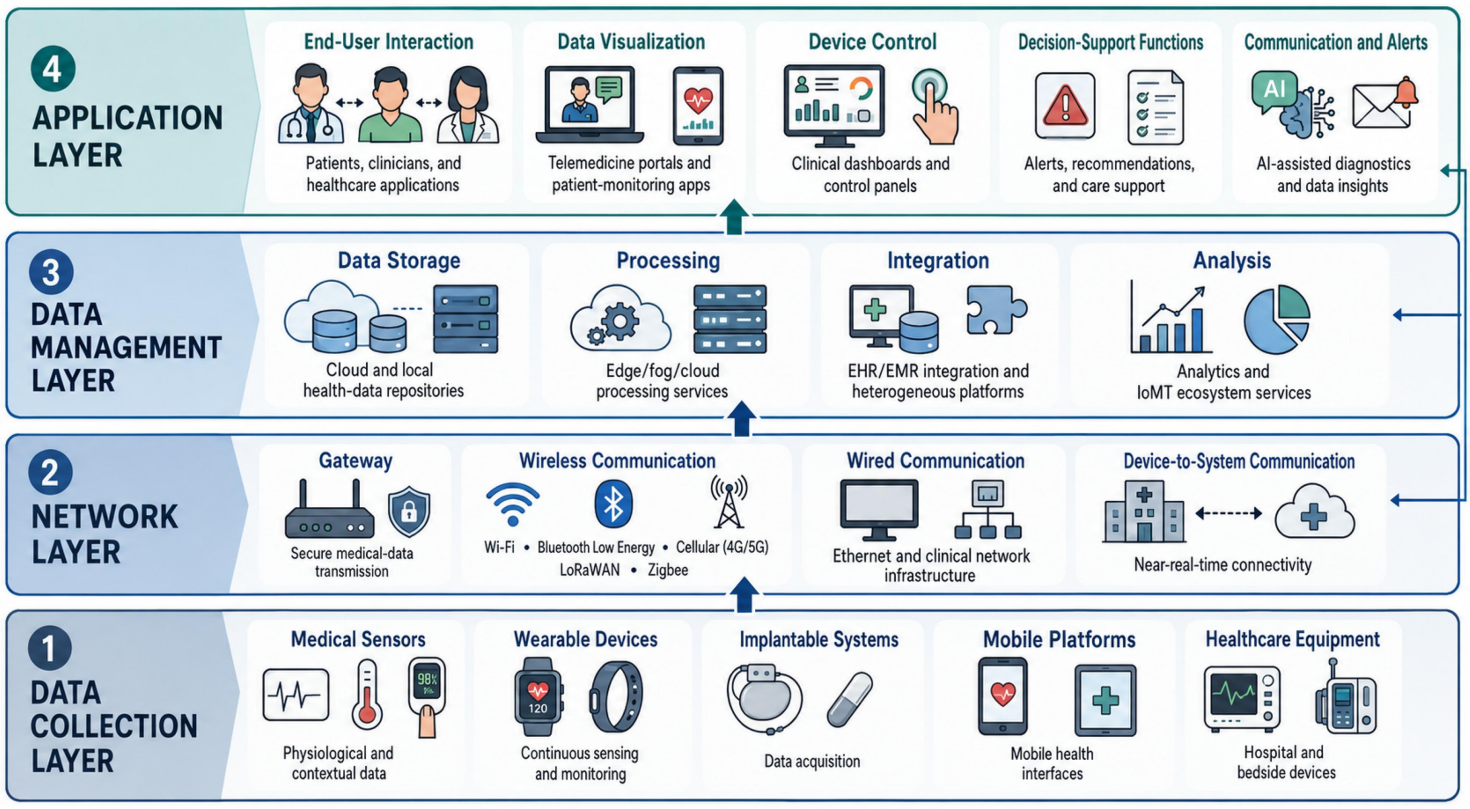
Layered IoMT architecture for connected healthcare.

The IoMT architecture can be conceptualized as a layered framework that connects patient-side sensing with network communication, data management, and clinical applications. Figure 1 summarizes this end-to-end structure.

The four layers are summarized as follows:

1. *Data collection layer:* This layer captures physiological, behavioural, and contextual data from medical sensors, wearable devices, implantable systems, mobile platforms, and healthcare equipment.
2. *Network layer:* This layer enables data transfer between devices, gateways, and higher-level services using wired and wireless technologies such as Wi-Fi, Bluetooth Low Energy, cellular networks, Zigbee, LoRaWAN, and Ethernet.
3. *Data management layer:* This layer supports storage, preprocessing, integration, analytics, and interoperability across heterogeneous devices, platforms, and clinical systems.
4. *Application layer:* This layer delivers user-facing functions such as telemedicine, patient monitoring, dashboards, alerts, device control, and clinical decision support.

This layered architecture is useful for reviewing neuro-fuzzy systems because deployment constraints differ across layers. Models operating near sensors or gateways must be lightweight and energy-aware, while cloud- or fog-based systems may support heavier analytics but can introduce latency, bandwidth, privacy, and interoperability concerns.

## 3 Neuro-fuzzy systems fundamentals

Neuro-fuzzy systems combine artificial neural networks with fuzzy logic to support learning from data while retaining a rule-based reasoning structure that can be inspected (15, 28). Neural components learn weights, tune membership functions, or optimize rule consequents, while fuzzy components represent uncertainty through linguistic variables, membership degrees, and interpretable rule structures. A fuzzy set assigns each input a degree of membership between 0 and 1, allowing clinical states such as low, moderate, or high risk to be represented gradually rather than as strict binary categories (14, 17).

In healthcare and IoMT settings, this integration is attractive because sensor and clinical data are frequently nonlinear, noisy, incomplete, heterogeneous, and temporally variable. NFS can support prediction, classification, monitoring, and decision support while preserving some traceability through fuzzy rules and membership functions (18). Common implementations include Adaptive Neuro-Fuzzy Inference Systems, fuzzy neural networks, deep neuro-fuzzy models, hybrid fuzzy-neural classifiers, and evolving neuro-fuzzy systems. These models differ in how rules are generated, how parameters are updated, and whether learning is static or adaptive.

The relevance of NFS to IoMT is therefore twofold. First, they can model uncertain and nonlinear patient data while supporting rule-based decision logic that may be interpretable when rule-base size, stability, fidelity, and readability are assessed. Second, their practical value depends on deployment characteristics such as rule-base size, update stability, latency, memory use, energy demand, privacy, and integration with clinical workflows. For this reason, the review treats NFS not only as predictive models but also as potential components of distributed connected-health systems.

## 4 Review of existing surveys on neuro-fuzzy systems in healthcare: gaps in the literature and study rationale

Recent advances in neuro-fuzzy systems, fuzzy logic, explainable artificial intelligence, edge intelligence, and Internet of Medical Things-enabled healthcare have generated a growing body of review literature. These reviews provide useful insights into diagnostic modelling, uncertainty handling, interpretability, intelligent sensing, and connected healthcare infrastructures. However, they do not yet provide a sufficiently focused and deployment-aware synthesis of neuro-fuzzy systems within Internet of Medical Things (IoMT) settings. In particular, existing reviews rarely examine neuro-fuzzy systems simultaneously across architectural paradigm, healthcare application domain, static or evolving learning behaviour, and deployment environment. This section reviews the most relevant survey literature, identifies its limitations, and establishes the rationale for the present review.

### 4.1 Recent surveys on neuro-fuzzy systems in healthcare

Several reviews have examined fuzzy and neuro-fuzzy models in healthcare, but their emphasis differs from the focus of the present review. Kour et al. (15) reviewed neuro-fuzzy systems for medical diagnosis and classification and reported strong predictive performance across several disease categories. However, the review focused mainly on diagnostic accuracy and classification performance, with limited attention to deployment-related issues such as latency, interoperability, resource constraints, edge or fog execution, and integration within connected-health infrastructures.

Abdalla et al. (19) reviewed fuzzy logic systems in Internet of Things-enabled smart healthcare using a PRISMA-based approach. That review is useful for understanding the broader role of fuzzy logic in healthcare applications, including smart monitoring and decision support. Nevertheless, neuro-fuzzy systems were treated as part of the wider fuzzy logic literature rather than as a distinct family of trainable models with potential rule-level transparency. Consequently, the review did not provide a dedicated taxonomy of neuro-fuzzy architectures, static and evolving learning behaviour, deployment layers, or deployment-sensitive evaluation metrics.

Jiang et al. (21) reviewed fuzzy intelligence and edge computing for health monitoring, with particular emphasis on infectious disease surveillance. Their work is relevant to deployment-aware healthcare analytics because it discusses latency reduction, privacy preservation, and edge-enabled processing. However, its application scope is relatively narrow and does not provide a broader synthesis of neuro-fuzzy system design across diverse IoMT healthcare applications, communication environments, and adaptive learning contexts.

Other reviews have focused on explainability and fuzzy reasoning in clinical decision support. Cao et al. (20) examined interpretable fuzzy inference systems for explainable diagnosis and contributed to the discussion of transparency in clinical decision-making. However, distributed deployment, interoperability, resource constraints, and real-time operational requirements were not treated as central review dimensions. Similarly, Zheng et al. (22) presented a taxonomy of fuzzy deep learning models for healthcare, with useful coverage of uncertain medical data, segmentation, and classification. However, that review gave limited attention to standalone neuro-fuzzy systems, evolving neuro-fuzzy models, and their feasibility under cloud, fog, edge, or hybrid IoMT deployment conditions.

Broader reviews of artificial intelligence and IoMT healthcare systems, including Manickam et al. (11), Arefin et al. (3), and He et al. (6), provide important context on connected healthcare architectures, sensing platforms, data analytics, and intelligent medical services. These reviews help explain the wider digital-health environment in which neuro-fuzzy systems may operate. However, because they address artificial intelligence and IoMT technologies more generally, they do not isolate neuro-fuzzy systems as a distinct methodological class. As a result, they provide limited article-level insight into how neuro-fuzzy models differ in terms of interpretability, adaptability, architectural design, deployment fidelity, and operational evaluation.

### 4.2 Motivation for the review

The comparison of selected background reviews highlights each review’s thematic focus and the extent to which it addresses deployment-aware, interoperability, or IoMT-specific considerations. These background reviews were used only to frame the research gap and were not included in the systematic evidence base.

The comparison shows that previous reviews tend to emphasize one of five perspectives: neuro-fuzzy diagnosis, fuzzy logic in smart healthcare, edge-enabled fuzzy intelligence, explainable fuzzy reasoning, or broad artificial-intelligence and IoMT healthcare systems. These perspectives are valuable, but they leave important gaps. First, neuro-fuzzy systems are often treated either as diagnostic tools or as a subset of general fuzzy logic rather than as a distinct model family that combines neural learning with rule-based fuzzy reasoning that can be evaluated for interpretability. Second, existing reviews rarely distinguish static neuro-fuzzy systems from evolving neuro-fuzzy systems, even though this distinction is important for continuous learning, concept-drift handling, and patient-specific adaptation in IoMT environments. Third, previous reviews provide limited article-level evidence on deployment environments, despite the direct influence of cloud, fog, and edge execution on latency, bandwidth use, privacy, energy consumption, scalability, and interoperability. Fourth, most reviews emphasize predictive performance or explainability, while deployment-aware evaluation metrics such as latency, memory use, energy consumption, communication overhead, throughput, robustness, and real-world integration are inconsistently reported or not treated as core review dimensions.

The present review addresses these limitations through a structured and deployment-aware synthesis of neuro-fuzzy systems in IoMT-enabled healthcare. Its central contribution is an article-level taxonomy that links neuro-fuzzy architecture, healthcare application domain, static or evolving design, and deployment environment within a single analytical framework. Unlike prior reviews, it distinguishes neural-network-based optimization, deep neuro-fuzzy systems, hybrid neuro-fuzzy systems, and evolving neuro-fuzzy systems; maps included peer-reviewed journal articles to predictive, detection, care-support, and recommender functions; and examines whether deployment-sensitive metrics such as latency, energy use, memory use, interoperability, and operational limitations are reported.

In addition, the present review provides Supplementary Table S1, which links each included article to its application area, data source, dataset type, neuro-fuzzy model, static or evolving classification, deployment layer, evaluation metrics, deployment-sensitive reporting, and main limitation. This improves traceability between individual included peer-reviewed journal articles and the aggregate synthesis. By combining a transparent screening protocol, a deployment-aware taxonomy, and a critical synthesis of operational gaps, the review provides a clearer foundation for adaptive, interpretability-aware, resource-aware, and clinically responsible neuro-fuzzy systems for future connected healthcare.

## 5 Methodology for the systematic review

This review was conducted using a structured systematic review methodology adapted from established review procedures in healthcare and Internet of Things research (29). The protocol was tailored to the specific context of neuro-fuzzy systems in the Internet of Medical Things, with emphasis on architectural paradigms, healthcare application domains, deployment environments, evaluation practices, and implementation constraints. The review process began on **6 March 2025** and ended with the final search and verification on **31 July 2025**; the same end date defined the upper bound of the publication window, which began on 1 January 2020. Searches were restricted to English-language peer-reviewed journal articles. The systematic evidence base comprised only peer-reviewed journal articles that satisfied the eligibility criteria. Non-journal sources, review articles, conference papers, books, book chapters, theses, preprints, editorials, commentaries, and similar materials were excluded from the study-selection counts, extraction matrix, quality assessment, taxonomy construction, and synthesis. Where such sources are cited elsewhere in the manuscript, they are used only for background, conceptual framing, methodological context, or technical explanation. The final synthesis included **55 peer-reviewed journal articles**. The complete article-level evidence base is provided in Supplementary Table S1.

**Figure 2:**
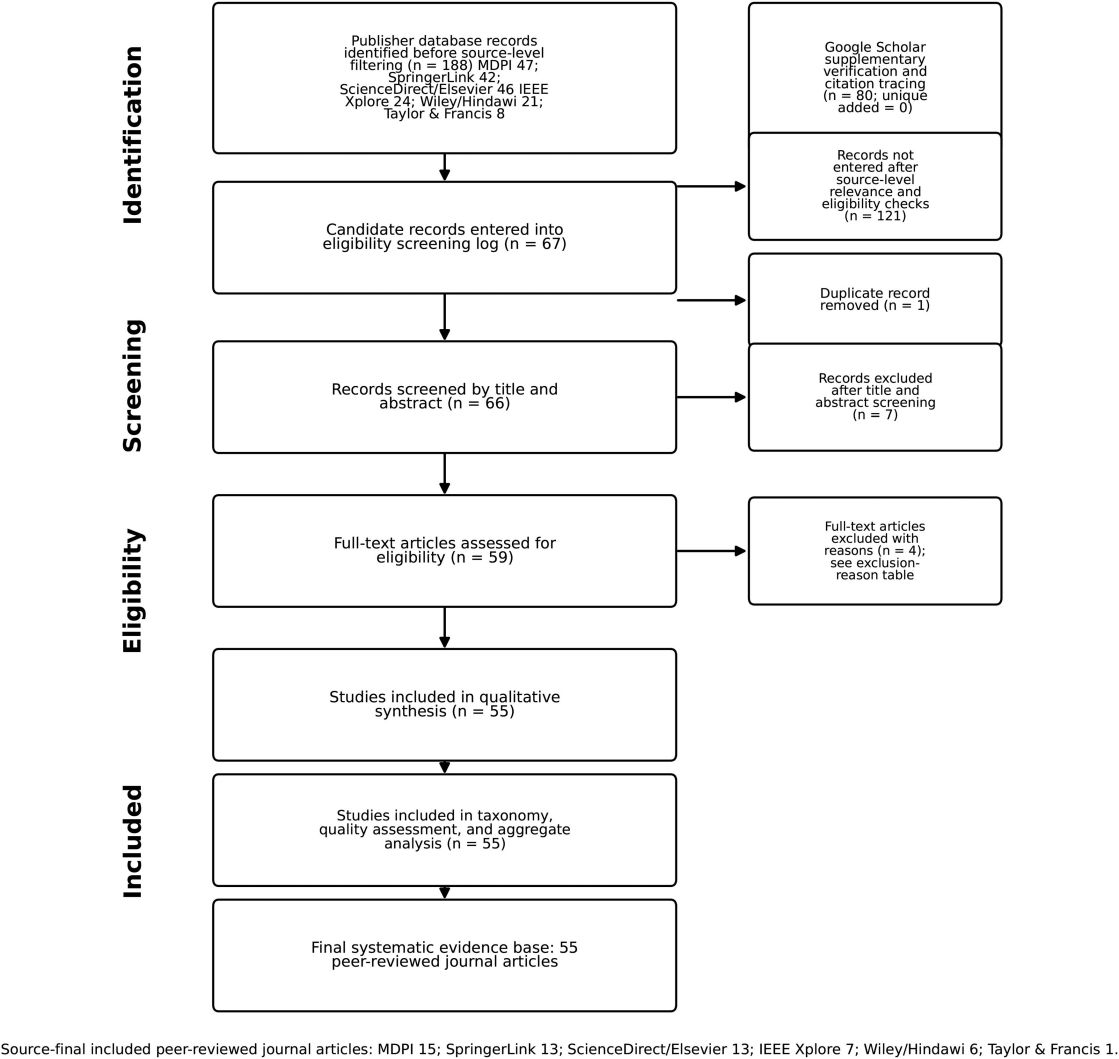
A flow diagram showing source-level record identification from publisher databases before source-level filtering, Google Scholar supplementary verification and citation tracing, duplicate removal, title and abstract screening, full-text eligibility assessment, exclusion reasons summarized in Table 5, and final inclusion of peer-reviewed journal articles only.

The review followed a systematic search and screening process in which each stage, from record identification and duplicate checking to screening, full-text assessment, exclusion recording, and final inclusion, was documented to ensure transparency and traceability. The process is summarised using a structured flow diagram in Figure 2. Search outputs, downloaded full-text articles, screening notes, and paper summaries were organized in a dedicated Google Drive repository maintained for the review. Instead of using a reference manager or systematic review platform, the review applied a standardized paper-summary rubric to document the screening and extraction process. For empirical peer-reviewed journal articles, the rubric captured article title, year, authors, journal, proposed method, research motivation, methodology, software tools, datasets, models, performance metrics, key results, limitations, and potential future research directions. For review papers identified during background screening, the rubric captured challenges related to the review scope, future research directions, and references for follow-up screening. This process provided an audit trail for identifying, screening, summarizing, and synthesizing the included peer-reviewed journal articles, while keeping background literature separate from the systematic evidence base.

### 5.1 Research framework and questions

The review process consisted of three phases: planning, conducting, and documenting. In the planning phase, the scope, research questions, search strategy, eligibility criteria, and extraction framework were defined. In the conducting phase, candidate records were identified, duplicates were removed, titles and abstracts were screened, full texts were assessed, and eligible peer-reviewed journal articles were retained. In the documenting phase, extracted evidence was synthesized according to architecture, application domain, deployment environment, evaluation practice, and open research issues.

The review was guided by five research questions. **RQ1** examined the practical motivations for adopting NFS in IoMT and connected healthcare. **RQ2** identified the healthcare application domains in which NFS have been implemented. **RQ3** classified NFS architectures across functional and deployment roles. **RQ4** examined evaluation metrics and methods used to assess NFS-IoMT evidence in the included peer-reviewed journal articles. **RQ5** identified trends, challenges, limitations, and future research directions. These questions informed the search strategy, extraction matrix, taxonomy, results analysis, and discussion.

### 5.2 Search strategy and information sources

A structured search strategy was used to identify peer-reviewed journal articles on neuro-fuzzy systems in Internet of Medical Things (IoMT)-enabled and connected-healthcare contexts. The strategy was designed to capture candidate records that combined a relevant neuro-fuzzy or fuzzy-neural method with a healthcare, medical-signal, clinical, sensor-based, IoMT, smart-healthcare, or connected-health deployment context. Three concept groups guided the search: (i) neuro-fuzzy or closely related fuzzy-neural methods, (ii) IoMT, medical IoT, smart-healthcare, sensor-based, or connected-healthcare context, and (iii) healthcare application, clinical task, or deployment-related terms. To improve reporting clarity and avoid errors caused by very long Boolean expressions, the search strategy was implemented as shorter search-string blocks rather than as a single combined query. Table 1 reports the S1-S9 search-word blocks used across the publisher databases. The blocks were used as base search expressions and were adapted where required to match each platform’s search interface, including differences in phrase handling, hyphenation, quotation marks, plural forms, wildcard support, and Boolean-operator behaviour. These syntax adjustments did not alter the eligibility criteria.

**Table 1:** S1–S9 search-word blocks used across publisher databases.

ID	Search focus	Method terms	IoMT/connected-health context terms	Application/deployment terms
S1	Core neuro-fuzzy IoMT	“neuro-fuzzy” OR “neuro fuzzy” OR “neuro-fuzzy system” OR “ANFIS” OR “adaptive neuro-fuzzy inference system”	“Internet of Medical Things” OR “IoMT” OR “medical IoT” OR “healthcare IoT”	“clinical decision support” OR “patient monitoring” OR “disease diagnosis” OR “predictive analytics”
S2	Connected healthcare	“neuro-fuzzy system” OR “neuro-fuzzy logic” OR “hybrid neuro-fuzzy” OR “fuzzy neural network”	“smart healthcare” OR “connected healthcare” OR “remote patient monitoring” OR “wearable health monitoring”	“health data analytics” OR “medical imaging” OR “patient monitoring” OR “disease prediction”
S3	ANFIS healthcare IoT	“ANFIS” OR “adaptive neuro-fuzzy inference system”	“healthcare IoT” OR “medical IoT” OR “smart healthcare” OR “connected healthcare”	“diagnosis” OR “classification” OR “prediction” OR “clinical decision support”
S4	Wearable and sensor-based healthcare	“neuro-fuzzy” OR “ANFIS” OR “adaptive neuro-fuzzy inference system”	“wearable health monitoring” OR “wearable devices” OR “wireless body sensor network” OR “wireless body area network”	“patient monitoring” OR “remote monitoring” OR “healthcare” OR “medical”
S5	Evolving fuzzy healthcare streams	“evolving fuzzy system” OR “evolving neuro-fuzzy system” OR “evolving fuzzy neural network”	“healthcare” OR “medical” OR “patient monitoring” OR “disease diagnosis”	“streaming data” OR “online learning” OR “concept drift” OR “adaptive learning”
S6	Deep and hybrid neuro-fuzzy healthcare	“deep neuro-fuzzy system” OR “hybrid neuro-fuzzy” OR “neuro-fuzzy logic” OR “fuzzy LSTM” OR “fuzzy BiLSTM” OR “fuzzy GRU” OR “fuzzy deep learning” OR “fuzzy deep network”	“healthcare IoT” OR “smart healthcare” OR “medical IoT” OR “connected healthcare”	“diagnosis” OR “prediction” OR “classification” OR “medical imaging”
S7	Deployment environment	“neuro-fuzzy” OR “ANFIS” OR “adaptive neuro-fuzzy inference system”	“healthcare” OR “medical” OR “IoMT” OR “Internet of Medical Things”	“edge computing” OR “fog computing” OR “cloud computing” OR “fog-cloud architecture”
S8	Resource-aware evaluation	“neuro-fuzzy” OR “ANFIS” OR “evolving neuro-fuzzy system”	“healthcare” OR “IoMT” OR “medical IoT” OR “smart healthcare”	“latency reduction” OR “resource optimization” OR “energy consumption” OR “memory” OR “throughput”
S9	Fuzzy reinforcement and adaptive-control healthcare	“fuzzy reinforcement learning” OR “fuzzy reinforcement” OR “fuzzy deep reinforcement learning” OR “fuzzy-based learning” OR “fuzzy intelligence” OR “fuzzy inference system”	“Internet of Medical Things” OR “IoMT” OR “healthcare IoT” OR “smart healthcare”	“clinical decision support” OR “patient monitoring” OR “resource optimization” OR “adaptive control” OR “anomaly detection” OR “security”

The blocks were designed to avoid two common retrieval problems. First, searches using only the exact phrase “Internet of Medical Things” were too narrow because many eligible peer-reviewed journal articles used related terms such as medical IoT, healthcare IoT, smart healthcare, wearable monitoring, physiological signals, edge computing, fog computing, or cloud-based healthcare. Second, searches using only broad terms such as healthcare and artificial intelligence were too general because they retrieved many general machine-learning, fuzzy-only, or clinical prediction records without a neuro-fuzzy or fuzzy-neural component. Each search block therefore combined a method component with a healthcare, IoMT, sensing, application, or deployment context where supported by the database interface. Where a platform did not support the full Boolean structure, the same logic was implemented through shorter phrase searches followed by manual eligibility screening.

The same search logic was applied across all publisher sources, with syntax adapted to each platform. MDPI, SpringerLink, ScienceDirect/Elsevier, IEEE Xplore, Wiley Online Library/Hindawi, and Taylor & Francis were searched using available journal-article filters, year limits, and field or metadata options. Searches were not restricted to health-only journals because relevant NFS–IoMT articles appeared across medical, engineering, computing, sensing, fuzzy-systems, and applied-science outlets. Supplementary Table S2 gives the platform-specific strings, fields, filters, dates, counts, and final source-attributed records. Searches were restricted to English-language peer-reviewed journal articles published within the defined review window. Google Scholar was used only for supplementary verification and citation tracing at the final verification stage. Where a record was available through a publisher platform, the publisher record was retained for source attribution. The search audit is reported at two levels. Table 2 gives the source-level summary, including search-block IDs, filters, retrieval and screening counts, full-text assessments, final included records, and source-attributed article identifiers. Supplementary Table S2 provides the complete audit, including exact platform-adapted strings, field settings, filters, dates, Google Scholar phrases, duplicate handling, and final inclusion counts.

**Table 2:** Source-level search summary and final included peer-reviewed journal-article identifiers.

Database/source	IDs	Platform filters and limits	Records retrieved	Records entered	Title/abstract retained	Full texts assessed	Included journal articles	Share	Final included article identifiers
MDPI	S1–S9	Article Type = Article where available; 2020–2025; all MDPI journals searched; English and journal-article eligibility checked manually	47	31	21	21	15	27.3%	(30, 33, 39, 40, 50, 54, 56, 60, 61, 66, 70, 71, 76, 83, 84)
SpringerLink	S1–S9	Journal articles; 2020–2025; English-language and eligibility limits checked through platform filters and manual screening	42	14	13	13	13	23.6%	(36, 37, 41, 42, 44, 45, 47, 51, 57, 67, 73, 77, 81)
ScienceDirect/Elsevier	S1–S9	Research articles; 2020–2025; English-language and journal-article status verified during screening	46	14	13	13	13	23.6%	(32, 53, 55, 62, 63, 65, 68, 72, 74, 75, 79, 80, 82)
IEEE Xplore	S1–S9	Journals; 2020–2025; English-language and eligibility limits verified manually during screening	24	8	7	7	7	12.7%	(34, 35, 38, 46, 52, 64, 78)
Wiley Online Library/Hindawi	S1–S9	Journal articles; 2020–2025; English-language and eligibility limits verified manually during screening	21	7	6	6	6	10.9%	(31, 43, 48, 49, 58, 59)
Taylor & Francis	S1–S9	Journal articles; 2020–2025; English-language and eligibility limits verified manually during screening	8	2	1	1	1	1.8%	(69)
**Publisher database total before source-level filtering**	–	–	**188**	–	–	–	–	–	MDPI 47; SpringerLink 42; ScienceDirect/Elsevier 46; IEEE Xplore 24; Wiley Online Library/Hindawi 21; Taylor & Francis 8.
Google Scholar	S1, S2, S5, S6, S7, S9	2020–2025; supplementary coverage check and citation tracing only; publisher records retained for source attribution where available	80	0	0	0	0	0.0%	No unique final source-attributed records.
**Final after filtering, duplicate checking, and screening**	–	–	–	**67**	**59**	**59**	**55**	**100%**	Final evidence base of 55 peer-reviewed journal articles.

Publisher searches were first conducted on 6 March 2025 and completed through the final verification stage on 31 July 2025. Google Scholar was used only for supplementary verification and citation tracing.

The bold values represent total number of articles retrieved across the publisher databases before the screening outcomes.

Google Scholar was handled separately from the publisher database searches because its ranking and result ordering may vary over time due to indexing updates, citation-based ranking, personalization, and search-result reordering. It was therefore not used as a primary database for final source attribution, but only as a supplementary verification source to check coverage and trace potentially relevant citations. To limit ranking variability, the Google Scholar verification procedure was fixed in advance. Eight phrase searches were used, and only the first results page was screened for each phrase, with a maximum screening window of 10 results per phrase. This produced 80 Google Scholar results screened in total. The exact phrases, screening window, duplicate handling, and outcomes are reported in Supplementary Table S2. Potentially relevant Google Scholar records were compared with the publisher-database screening log using article title, DOI, author names, publication year, and journal or publisher source. Where a Google Scholar result was also available through a publisher platform, the publisher record was retained for source attribution. No included peer-reviewed journal article was identified only through Google Scholar, and Google Scholar contributed no unique record to the final 55 peer-reviewed journal-article evidence base.

Records retrieved refers to the results returned after applying the stated search blocks and platform filters. The publisher searches returned 188 records before source-level relevance and eligibility checks. After removing records that were duplicate, out of scope, non-journal, background-only, or clearly ineligible, 67 candidate records entered the screening log. Title and abstract screening retained 59 records for full-text assessment, and 55 peer-reviewed journal articles were included in the final evidence base. Google Scholar was screened separately for verification and citation tracing; 80 results were checked, but no unique publisher-attributed record was added. Supplementary Table S2 provides the audit trail supporting these counts. IoMT relevance was assessed using both explicit terminology and contextual evidence. Broader connected-health terms, including medical IoT, healthcare IoT, smart healthcare, remote patient monitoring, wearable health monitoring, wireless body sensor networks, physiological signals, edge computing, fog computing, and cloud computing, were used to improve retrieval sensitivity because relevant peer-reviewed journal articles do not always use the exact phrase “Internet of Medical Things.” However, these broader terms did not determine final inclusion.

To limit over-inclusion of general healthcare AI studies, the screening process applied a two-part specificity check. First, the article had to report a neuro-fuzzy, Adaptive Neuro-Fuzzy Inference System, evolving fuzzy, fuzzy-neural, deep neuro-fuzzy, hybrid neuro-fuzzy, or closely related fuzzy-inference method. Second, the healthcare setting had to show a connected-health or IoMT-relevant context. Eligible contextual evidence included wearable or physiological sensor data, wireless body area networks, remote patient monitoring, smart-healthcare infrastructure, connected medical devices, or edge-, fog-, or cloud-supported health monitoring. Articles were excluded if they used general artificial-intelligence or machine-learning methods without a neuro-fuzzy or fuzzy-neural component. Healthcare AI articles were also excluded if they addressed diagnosis, prediction, image analysis, or clinical decision support without a clear sensor-based, networked, remote-monitoring, or connected-health component. This approach allowed the search to remain sensitive to varied terminology while keeping the final evidence base specific to NFS-IoMT and connected-health applications.

### 5.3 Study selection

Study selection was performed in four stages: identification, duplicate removal, title and abstract screening, and full-text eligibility assessment. The publisher database searches identified 188 records before source-level filtering. Source-level relevance and eligibility checks removed 121 records that were duplicates within source outputs, outside the review scope, non-journal records, background-only records, or otherwise unsuitable for eligibility screening. The remaining 67 candidate records were entered into the screening log. Duplicates were removed manually using bibliographic matching based on article title, author names, publication year, DOI, and publication source. The remaining records were screened by title and abstract against the predefined inclusion and exclusion criteria. Articles considered potentially relevant proceeded to full-text assessment. An article was retained only if it satisfied all inclusion criteria and did not meet any exclusion criterion. Google Scholar was used separately for supplementary verification and citation tracing; it did not add a unique source-attributed record to the final evidence base.

Screening, data extraction, and quality assessment followed a verification-based two-author process. The first author performed the initial title and abstract screening, full-text eligibility assessment, data extraction, and quality scoring, while the second author independently verified the screening decisions, extracted information, and assigned scores. Formal inter-rater agreement statistics were not calculated because the process was based on first-author assessment followed by second-author verification rather than fully independent parallel screening. This is acknowledged as a methodological limitation.

The eligibility criteria are presented in Table 3. Only peer-reviewed journal articles reporting empirically evaluated neuro-fuzzy systems in Internet of Medical Things-related or connected-healthcare contexts were included in the systematic evidence base. Review papers, conference papers, short papers, non-English publications, books, book chapters, commentaries, editorials, theses, preprints, and non-peer-reviewed materials were excluded from the final systematic evidence base. Such materials, where cited, were used only as background or contextual references and were not included in the study-selection counts, extraction matrix, quality assessment, taxonomy construction, or synthesis.

**Table 3:** Inclusion and exclusion criteria for selecting peer-reviewed journal articles for the systematic evidence base.

Inclusion criteria	Exclusion criteria
**IC1.** Peer-reviewed journal articles presenting neuro-fuzzy system applications in Internet of Medical Things-related or connected-healthcare contexts, including disease detection, disease prediction, patient monitoring, clinical decision support, care-support functions, or recommender functions.	**EC1.** Articles that did not explicitly involve neuro-fuzzy, Adaptive Neuro-Fuzzy Inference System, evolving fuzzy, deep neuro-fuzzy, hybrid neuro-fuzzy, or closely related neuro-fuzzy methods in healthcare or Internet of Medical Things-related domains.
**IC2.** Articles published in English within the defined review window, from 1 January 2020 through the final search date.	**EC2.** Articles that lacked an Internet of Medical Things-related, connected-health, sensor-based, wearable, smart-healthcare, remote-monitoring, or distributed healthcare-computing context.
**IC3.** Articles published as peer-reviewed journal articles in recognized scholarly databases or publisher platforms.	**EC3.** Books, book chapters, conference papers, symposium papers, workshop papers, theses, preprints, review articles, commentaries, editorials, letters, perspective pieces, and non-English publications.
**IC4.** Articles reporting empirical validation, including experimental evaluation, simulation under representative conditions, benchmarking against baseline methods, or real-world deployment evidence.	**EC4.** Articles shorter than six pages, inaccessible full texts, duplicate publications, articles outside the eligible date range, or articles without sufficient methodological and evaluation detail.
**IC5.** Articles providing sufficient information on the neuro-fuzzy model, dataset, evaluation setting, and reported performance metrics to support extraction and comparison.	**EC5.** Articles lacking sufficient information for classification within the architecture, application-domain, deployment, or evaluation taxonomy.

The study-selection numerical summary is presented in Table 4, and the corresponding flow diagram is shown in Figure 2.

**Table 4:** Study-selection summary for the 55 peer-reviewed journal articles included in the systematic evidence base.

Selection stage	Records	Clarification
Records identified from publisher databases before source-level filtering	188	MDPI 47; SpringerLink 42; ScienceDirect/Elsevier 46; IEEE Xplore 24; Wiley Online Library/Hindawi 21; Taylor & Francis 8.
Records not entered after source-level relevance and eligibility checks	121	Removed before the eligibility screening log because records were duplicates within source outputs, outside scope, non-journal records, background-only records, or otherwise failed preliminary relevance and eligibility checks.
Candidate records entered into the eligibility screening log	67	Unique candidate records retained for formal eligibility screening.
Duplicate record removed	1	Duplicate removed using bibliographic matching.
Records screened by title and abstract	66	Records assessed against the predefined inclusion and exclusion criteria.
Records excluded after title and abstract screening	7	Excluded before full-text eligibility assessment.
Full-text articles assessed for eligibility	59	Full texts assessed against all inclusion and exclusion criteria.
Full-text articles excluded with reasons	4	Exclusion reasons are summarized by screening stage in Table 5.
Peer-reviewed journal articles included in the qualitative synthesis	55	Peer-reviewed journal articles retained in the final synthesis.
Peer-reviewed journal articles included in taxonomy, quality assessment, and aggregate analysis	55	Same final evidence base used for taxonomy, quality appraisal, and aggregate reporting.
Final systematic evidence base	55	Peer-reviewed journal articles.
Google Scholar supplementary verification and citation tracing results screened	80	Used only for supplementary coverage checking and citation tracing.
Unique records added from Google Scholar to the publisher-attributed evidence base	0	No unique source-attributed final record was added from Google Scholar.

The exclusion-reason counts in Table 5 refer only to records excluded after title/abstract screening and full-text eligibility assessment. The duplicate record removed before screening is reported separately in Table 4 and Figure 2 and is therefore not included as an exclusion reason. Accordingly, the exclusion-reason table reconciles with the screening pathway: 7 records were excluded after title/abstract screening and 4 full-text articles were excluded after eligibility assessment.

**Table 5:** Reasons for exclusion during title/abstract screening and full-text eligibility assessment. Excluded reviews, conference papers, books, and other non-journal sources were not counted in the systematic evidence base.

Exclusion reason	Title/abstract stage	Full-text stage
Review, survey, editorial, commentary, or background-only article	3	1
Outside the eligible publication period or not a peer-reviewed journal article	1	0
Fuzzy-only, machine-learning-only, or artificial-intelligence study without a neuro-fuzzy or fuzzy-neural component	2	1
General healthcare AI article without a clear IoMT, connected-health, sensor-based, wearable, remote-monitoring, or distributed healthcare-computing context	1	1
Insufficient methodological, evaluation, or extraction detail for taxonomy coding	0	1
**Total excluded**	**7**	**4**

The bold values represent the total number of records excluded after Title/abstract and Full-text stages.

### 5.4 Final included peer-reviewed journal articles

The final systematic evidence base included 55 peer-reviewed journal articles published between 2020 and 2025. These articles were the only records included in the selection counts, extraction matrix, quality appraisal, taxonomy, and quantitative synthesis. The final included articles were: (30–84).

Supplementary Table S1 provides the complete extraction matrix for the 55 included peer-reviewed journal articles. It reports article details, application domain, architecture, dataset, evaluation setting, deployment environment, performance results, deployment-sensitive reporting, quality scores, NFS-IoMT evidence-quality category, and primary contribution. It also includes source distribution, selection counts, exclusion reasons, coding definitions, verification notes, and the article-level quality appraisal.

### 5.5 Quality assessment

Each included peer-reviewed journal article was assessed across three NFS-IoMT evidence-quality dimensions: dataset adequacy, benchmarking rigor, and deployment fidelity. These dimensions capture empirical reliability, comparative strength, and relevance to IoMT deployment. Each dimension was scored as 0 = not addressed, 1 = partially addressed, or 2 = clearly addressed, giving an aggregate score from 0 to 6. Table 6 summarizes the rubric. Dataset adequacy covered source, size, representativeness, and clinical relevance. Benchmarking rigor covered relevant baselines and suitable metrics. Deployment fidelity covered IoMT-relevant conditions such as distributed execution, streaming data, latency, resource use, interoperability, privacy, and related deployment constraints.

**Table 6:** Quality assessment rubric applied only to the included peer-reviewed journal articles.

Dimension	Score	Interpretation
Dataset adequacy	0	Dataset source, data modality, size, or clinical relevance not addressed sufficiently for appraisal.
Dataset adequacy	1	Dataset or data modality reported, but with limited detail on size, provenance, representativeness, or clinical relevance.
Dataset adequacy	2	Dataset clearly described, clinically relevant or benchmarked, and adequate for the stated task.
Benchmarking rigor	0	No meaningful comparison with baseline methods or incomplete metric reporting.
Benchmarking rigor	1	Partial comparison with selected baselines or limited evaluation metrics.
Benchmarking rigor	2	Clear comparison against relevant baselines with appropriately reported metrics.
Deployment fidelity	0	No realistic deployment context or operational constraints considered.
Deployment fidelity	1	Partial consideration of practical factors such as latency, streaming, interoperability, privacy, or resource limits.
Deployment fidelity	2	Evaluation reflects realistic Internet of Medical Things conditions, such as distributed execution, streaming data, latency-aware processing, resource-aware operation, or real-world deployment.

The quality scores were not used as mechanical exclusion thresholds. Study selection was determined by the predefined eligibility criteria, while quality assessment was conducted after inclusion to support interpretation of the strength and limitations of the evidence for the review’s NFS-IoMT synthesis. Therefore, an article could remain in the review even if it received a low aggregate score, provided that it met the inclusion criteria. The low, moderate, and high categories refer to NFS-IoMT evidence quality within this review, especially dataset adequacy, benchmarking relevance, and deployment fidelity. They should not be interpreted as an overall quality judgement on the original article or on its contribution to the broader neuro-fuzzy systems field outside the specific NFS-IoMT evidence-synthesis criteria used in this review. Initial scoring was performed by the first author and independently verified by the second author. Where differences in interpretation arose, both authors discussed the scoring decision until consensus was reached. The final quality appraisal showed that aggregate scores ranged from 2 to 6, with a mean score of 3.98. Using the predefined interpretation bands, 8 included peer-reviewed journal articles were rated as low NFS-IoMT evidence quality (0–2), 26 as moderate NFS-IoMT evidence quality (3–4), and 21 as high NFS-IoMT evidence quality (5–6). No included article received an aggregate score of 0 or 1 after consensus scoring. This indicates that all included peer-reviewed journal articles provided at least some empirical evidence relevant to the review, although the depth of dataset reporting, benchmarking rigor, and deployment fidelity varied substantially across the evidence base.

Deployment fidelity was the weakest and most variable quality-assessment domain. Thirty-two included peer-reviewed journal articles received a deployment-fidelity score of 0, indicating that realistic deployment context or operational constraints were not reported. One article received a score of 1, reflecting partial deployment-sensitive evidence, while 22 articles received a score of 2 because they reported deployment-sensitive evidence such as execution time, latency, resource use, streaming considerations, or edge, fog, or cloud implementation details. Consequently, claims from articles with low deployment-fidelity scores were interpreted cautiously when discussing real-world IoMT readiness. Articles with stronger deployment-fidelity scores were given greater interpretive weight in the synthesis of practical deployability, runtime feasibility, and edge/fog/cloud suitability. The complete article-level quality appraisal is reported in Supplementary Table S1 together with the extraction matrix. For each included peer-reviewed journal article, Supplementary Table S1 reports the dataset adequacy score, benchmarking rigor score, deployment fidelity score, aggregate quality score, NFS–IoMT evidence-quality category, and a brief justification for the assigned scores. The scores were used to support comparative interpretation across the evidence base, particularly when discussing the gap between model-level performance and deployment-aware evaluation in Internet of Medical Things settings, and were not used as mechanical exclusion criteria or as general judgements of article quality outside the scope of this review.

### 5.6 Data extraction

A standardized data extraction form was used to record information from each included peer-reviewed journal article. The extracted fields comprised bibliographic information, application characteristics, architectural characteristics, deployment characteristics, evaluation characteristics, operational considerations, and quality assessment scores. Specifically, the following information was extracted:

1. bibliographic information: authors, year, title, journal, DOI, and publication source;
2. application characteristics: healthcare domain, clinical task, and Internet of Medical Things use case;
3. architectural characteristics: neuro-fuzzy system type, learning paradigm, adaptation capability, and model role;
4. deployment characteristics: cloud, fog, edge, hybrid, or unspecified execution context;
5. evaluation characteristics: datasets, baseline methods, validation strategy, and reported metrics;
6. implementation and operational aspects: latency, resource use, memory considerations, interoperability, privacy, security, or reported deployment limitations;
7. quality assessment scores and category: dataset adequacy, benchmarking rigor, deployment fidelity, aggregate score, and NFS–IoMT evidence-quality category.

This extraction scheme enabled structured comparison across included peer-reviewed journal articles and direct mapping to the review questions. The complete list of the 55 included peer-reviewed journal articles and their extracted characteristics is provided in Supplementary Table S1. Aggregate percentages reported in the synthesis were calculated using this denominator of 55.

### 5.7 Data synthesis

Following extraction, the included peer-reviewed journal articles were synthesized across three primary dimensions: neuro-fuzzy system architecture, application domain, and deployment environment. Architectural synthesis considered the type of neuro-fuzzy model, whether the model was static or adaptive, and the learning paradigm employed. Application synthesis grouped articles according to healthcare role, such as diagnosis, prediction, monitoring, care support, or recommendation. Deployment synthesis examined whether execution was performed in cloud, fog, edge, hybrid, or unspecified settings.

Where quantitative evidence was available, reported performance metrics such as accuracy, precision, recall, F1-score, latency, and resource use were compared across included peer-reviewed journal articles. Because the included peer-reviewed journal articles varied substantially in datasets, tasks, and evaluation settings, the synthesis was narrative and comparative rather than meta-analytic. This approach was considered more appropriate given the methodological heterogeneity of the included evidence base.

For percentage-based reporting, the denominator was the final evidence base of 55 included peer-reviewed journal articles. Each included peer-reviewed journal article was assigned one primary architecture category, one primary application-domain category, one primary medical-domain category, and one primary deployment category. Architecture classification was based on the dominant neuro-fuzzy model design, application-domain classification was based on the main healthcare function addressed, medical-domain classification was based on the principal disease or healthcare context, and deployment classification was based on the reported execution environment. Multi-label classification was not used for the aggregate taxonomy percentages; therefore, the reported architecture, application-domain, medical-domain, and deployment percentages sum to 100%, subject only to rounding. Where included peer-reviewed journal articles described multiple technical components, diseases, application functions, or deployment layers, the primary category reflected the main contribution and implementation context, while additional details were retained in Supplementary Table S1. Latency, energy, memory, and performance-metric reporting were coded separately as descriptive evidence fields rather than as mutually exclusive taxonomy categories; their percentages also use 55 as the denominator but are not expected to sum to 100% because an article could report more than one metric.

### 5.8 Documentation and reporting

The extracted and synthesized data were organized to align directly with the five research questions. Observations were classified according to architectural characteristics, healthcare application domains, deployment tiers, evaluation practices, and reported limitations. Recurring themes, evidence gaps, and methodological weaknesses were identified and incorporated into the final analysis. The results are reported using structured tables, comparative visualizations, taxonomy-based synthesis, a supplementary extraction matrix, a study-selection summary, an exclusion-reason table separated by title/abstract and full-text stage, and a structured flow diagram to support transparency, traceability, and future reuse in subsequent research on neuro-fuzzy systems for intelligent connected healthcare.

## 6 Taxonomy of neuro-fuzzy systems in internet of medical things

**Figure 3:**
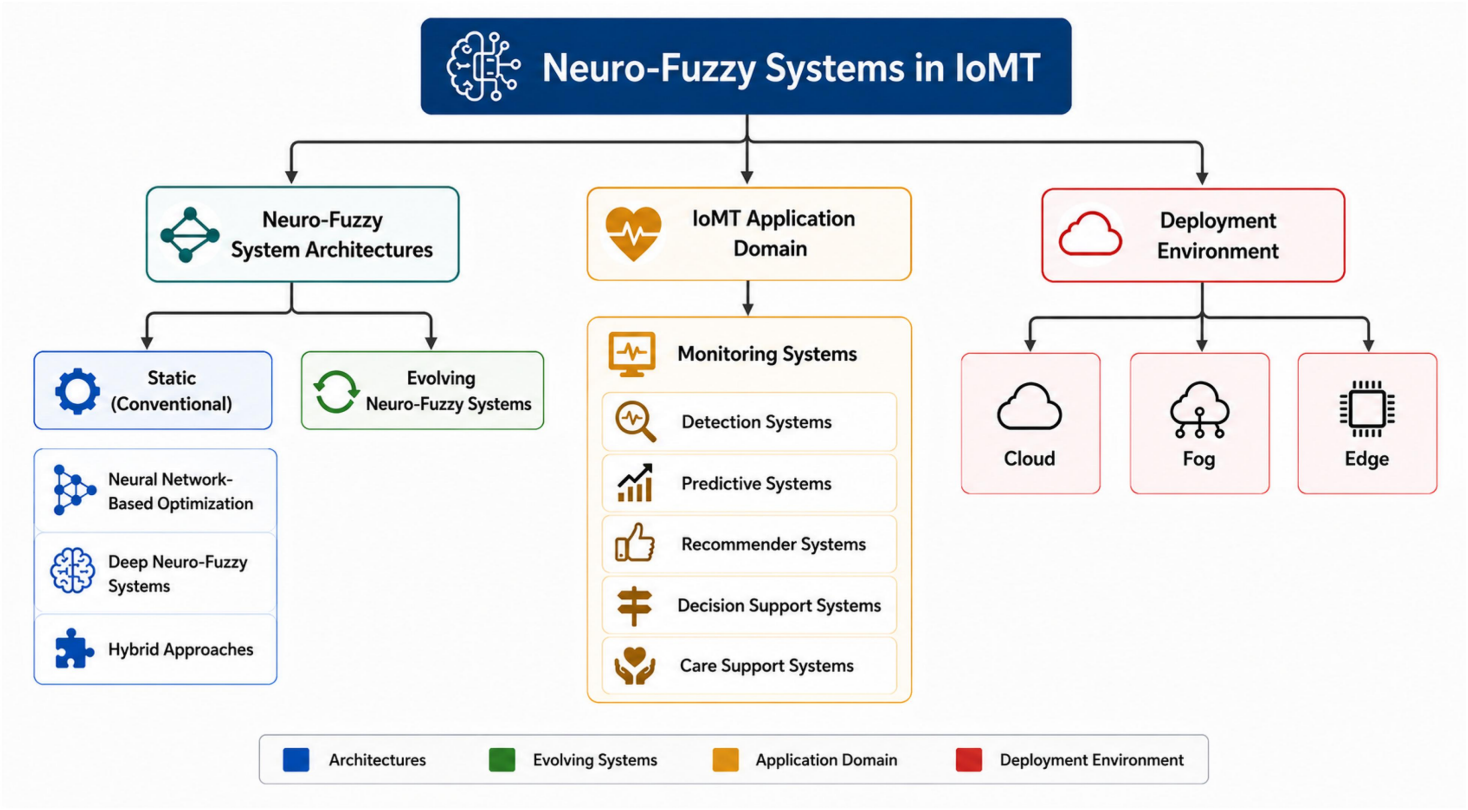
Taxonomy of neuro-fuzzy systems in Internet of Medical Things-enabled healthcare, based only on the 55 peer-reviewed journal articles included in the systematic evidence base.

This section reviews the 55 peer-reviewed journal articles included in the final systematic evidence base and proposes a structured taxonomy of neuro-fuzzy systems in Internet of Medical Things-enabled healthcare. The taxonomy is organized around three core dimensions: neuro-fuzzy system architecture, healthcare application domain, and deployment environment. As shown in Figure 3, this classification captures the main architectural, functional, and deployment-related characteristics of the included peer-reviewed journal articles, thereby providing a coherent basis for analysing neuro-fuzzy intelligence in connected healthcare. The article-level evidence supporting this taxonomy is provided in Supplementary Table S1.

The first dimension, **Neuro-fuzzy System Architecture**, captures both the learning behaviour and the dominant model design. Based on the article-level coding, static systems dominate the evidence base, accounting for 48 of the 55 included peer-reviewed journal articles (87.3%), while evolving systems account for 7 articles (12.7%). At the architectural-category level, neural-network-based optimization is the largest group, with 28 articles (50.9%), followed by hybrid neuro-fuzzy systems with 11 articles (20.0%), deep neuro-fuzzy systems with 9 articles (16.4%), and evolving neuro-fuzzy systems with 7 articles (12.7%). This distribution shows that most current work remains oriented toward offline model optimization and fixed inference structures, despite the dynamic and non-stationary nature of many IoMT environments.

The second dimension, **Internet of Medical Things Application Domain**, classifies neuro-fuzzy systems according to their main healthcare function. The included peer-reviewed journal articles are concentrated mainly in predictive and detection tasks. Predictive systems account for 29 articles (52.7%), detection systems account for 24 articles (43.6%), while care-support systems and recommender systems each account for 1 article (1.8%). This distribution indicates that current research is strongly oriented toward disease prediction, diagnostic classification, and abnormality detection, with limited evidence on longitudinal care support and personalized treatment recommendation.

The third dimension, **Deployment Environment**, captures where the models are positioned across cloud, fog-cloud, edge-cloud, or unspecified settings. Deployment reporting remains limited. Thirty-four included peer-reviewed journal articles (61.8%) did not clearly specify the deployment layer in the submitted manuscript or extraction table. Among articles that did report a deployment context, 15 articles (27.3%) used cloud or cloud-based Internet of Things or Internet of Medical Things settings, 5 articles (9.1%) used fog-cloud or fog-oriented settings, and 1 article (1.8%) used an edge-cloud setting. This distribution indicates that, although deployment environment is central to IoMT readiness, most included peer-reviewed journal articles remain primarily algorithm-focused and do not provide sufficient operational deployment detail.

### 6.1 Neuro-fuzzy system architecture

Neuro-fuzzy architectures reported in IoMT-oriented healthcare studies can be broadly classified as static or evolving. Static neuro-fuzzy systems are trained offline and retain fixed structures and parameters during inference (14, 18). Evolving neuro-fuzzy systems, on the other hand, update their parameters, rule bases, or structural components incrementally in response to incoming data (18, 85). This distinction is important because healthcare environments involve heterogeneous sensing platforms, temporally changing physiological signals, patient variability, and non-stationary operating conditions. As a result, architectural choice has direct implications for adaptability, computational cost, deployment feasibility, and long-term clinical utility.

#### 6.1.1 Static neuro-fuzzy systems

Static neuro-fuzzy systems represent the dominant learning pattern in the included evidence base, accounting for 48 of the 55 peer-reviewed journal articles (87.3%). These models are usually developed using historical or batch datasets, and their fuzzy rule bases, membership functions, and network weights remain fixed during inference (86). Their relative simplicity, potential rule-level transparency, and ease of implementation make them attractive for healthcare applications where the relationship between inputs and outputs is assumed to remain stable. However, their main limitation is the inability to adapt to concept drift, patient-specific variability, changing sensor conditions, or evolving clinical contexts without retraining.

Within the included evidence base, static neuro-fuzzy systems are mainly represented by neural-network-based optimization, deep neuro-fuzzy systems, and hybrid neuro-fuzzy systems.

##### 6.1.1.1 Neural-Network-Based Optimization

Neural-network-based optimization is the largest architectural category, comprising 28 of the 55 included peer-reviewed journal articles (50.9%). These systems use neural learning or metaheuristic optimization to tune fuzzy membership functions, rule bases, feature-selection procedures, or consequent parameters while retaining the uncertainty-handling capacity of fuzzy inference (87). Reported optimization strategies include genetic algorithms (30, 31, 54), particle-swarm-based and population-based optimization methods (35, 57), and broader metaheuristic or feature-selection approaches (34, 36, 44).

These models are used across cardiovascular, diabetes, Parkinson’s disease, cancer, glaucoma, remote monitoring, autism, and stress-related tasks (34, 35, 37, 45, 48, 49, 53, 56). Some articles mention cloud, fog, or hybrid fog-cloud execution, but detailed runtime evidence remains limited (38, 46, 47, 51).

Overall, this category demonstrates strong model-level performance across prediction and detection tasks, but most included peer-reviewed journal articles remain static, offline, and benchmark-oriented. Although some included articles mention fog or cloud deployment, detailed evaluation of real-time streaming, communication overhead, energy consumption, memory use, and sustained deployment performance remains limited.

##### 6.1.1.2 Deep Neuro-fuzzy Systems

Deep neuro-fuzzy systems account for 9 of the 55 included peer-reviewed journal articles (16.4%). These models combine the feature-extraction capability of deep learning with fuzzy inference (14, 86). Convolutional or recurrent networks are typically used to extract high-level representations from medical images, physiological signals, or multimodal data, after which fuzzy inference supports classification, prediction, or rule-based decision modelling.

The included deep neuro-fuzzy articles cover medical imaging, connected-health risk prediction, leukemia, kidney disease, cardiovascular diagnosis, Alzheimer’s disease, and Parkinson’s disease screening (58–61, 63–65).

These included articles show that deep neuro-fuzzy systems are relevant for high-dimensional, noisy, and multimodal health data. Their main strength lies in combining representation learning with fuzzy reasoning. However, most remain static and are evaluated offline. Cloud-centric execution is common, and resource-aware evaluation is rarely reported in detail, limiting the ability to judge their suitability for real-time IoMT deployment.

##### 6.1.1.3 Hybrid Neuro-fuzzy Systems

Hybrid neuro-fuzzy systems account for 11 of the 55 included peer-reviewed journal articles (20.0%). These systems integrate fuzzy inference with additional computational paradigms such as deep learning, ensemble learning, regression trees, federated learning, or metaheuristic optimization. The purpose is to combine complementary strengths in feature extraction, classification, optimization, privacy preservation, and decision fusion while retaining fuzzy reasoning structures that may support interpretability when evaluated.

Representative examples include recurrent neuro-fuzzy mining, deep and metaheuristic cancer detection, fusion-based stress recognition, federated fuzzy ensembles, neuro-fuzzy regression trees, arrhythmia ensembles, and IoT-enabled heart disease monitoring (67, 68, 70, 72–76).

The hybrid category broadens the neuro-fuzzy design space and often reports high classification performance. However, many models are computationally intensive, static, and evaluated on curated offline datasets. Even where real-time sensing, cloud infrastructure, or federated learning is mentioned, deployment-sensitive evidence remains limited.

#### 6.1.2 Evolving neuro-fuzzy systems

Evolving neuro-fuzzy systems account for 7 of the 55 included peer-reviewed journal articles (12.7%). These systems differ from static architectures because they support online learning, structural plasticity, and incremental rule adaptation (18, 78, 88). Rather than relying only on offline retraining, evolving systems update their internal structures and parameters as new observations arrive, making them conceptually well aligned with non-stationary IoMT environments.

Representative evolving articles address electroencephalography analysis, seismocardiogram artefact removal, heart sound classification, wearable biofeedback, stress detection from wearable IoMT data, cervical cancer diagnosis, and heart sound identification (78–84).

The reviewed evolving systems are more closely aligned with the adaptive and continuous-monitoring requirements of connected healthcare than static models. They support incremental learning and rule evolution, but their rule stability, auditability, and clinician readability still need explicit evaluation. Their practical and clinical readiness also remains unproven because deployment validation is limited, datasets are often narrow, and reporting of energy use, memory footprint, communication overhead, and sustained performance under realistic streaming conditions remains weak.

### 6.2 Neuro-fuzzy system application domain in internet of medical things

The application space of neuro-fuzzy systems in IoMT-enabled healthcare spans multiple functions, ranging from early prediction and real-time detection to longitudinal care support and personalized recommendation. The proposed application-domain taxonomy distinguishes between monitoring systems and recommender systems, following the general classification logic in (29). Monitoring systems are further divided into predictive systems, detection systems, and care-support systems.

Based on the 55 peer-reviewed journal articles in the systematic evidence base, predictive systems are the largest category, with 29 articles (52.7%). Detection systems form the second largest category, with 24 articles (43.6%). Care-support systems and recommender systems remain sparsely represented, with 1 article each (1.8%). This uneven distribution shows that current neuro-fuzzy research in IoMT-enabled healthcare is concentrated mainly on prediction and detection, while clinically important functions such as continuous care support and treatment recommendation remain underdeveloped.

#### 6.2.1 Predictive systems

Predictive systems are designed to forecast patient states, disease progression, or adverse clinical events before they occur. They typically learn from historical records, physiological measurements, or continuously generated health data to support early intervention and preventive care (29). In neuro-fuzzy systems, fuzzy reasoning captures uncertainty and nonlinear clinical relationships, while neural learning supports data-driven tuning of decision boundaries (89).

The included predictive articles are concentrated mainly in cardiovascular, metabolic, neurological, mental-health, and multi-disease prediction tasks. Cardiovascular prediction is the most prominent area, with articles addressing heart disease diagnosis, wearable heart monitoring, and cardiovascular risk assessment (38, 43, 48, 50, 66, 67, 69, 72, 77). Diabetes prediction forms another major cluster, with cloud, fog-cloud, and benchmark-based Adaptive Neuro-Fuzzy Inference System formulations reporting strong results (39, 46, 51, 56).

**Figure 4:**
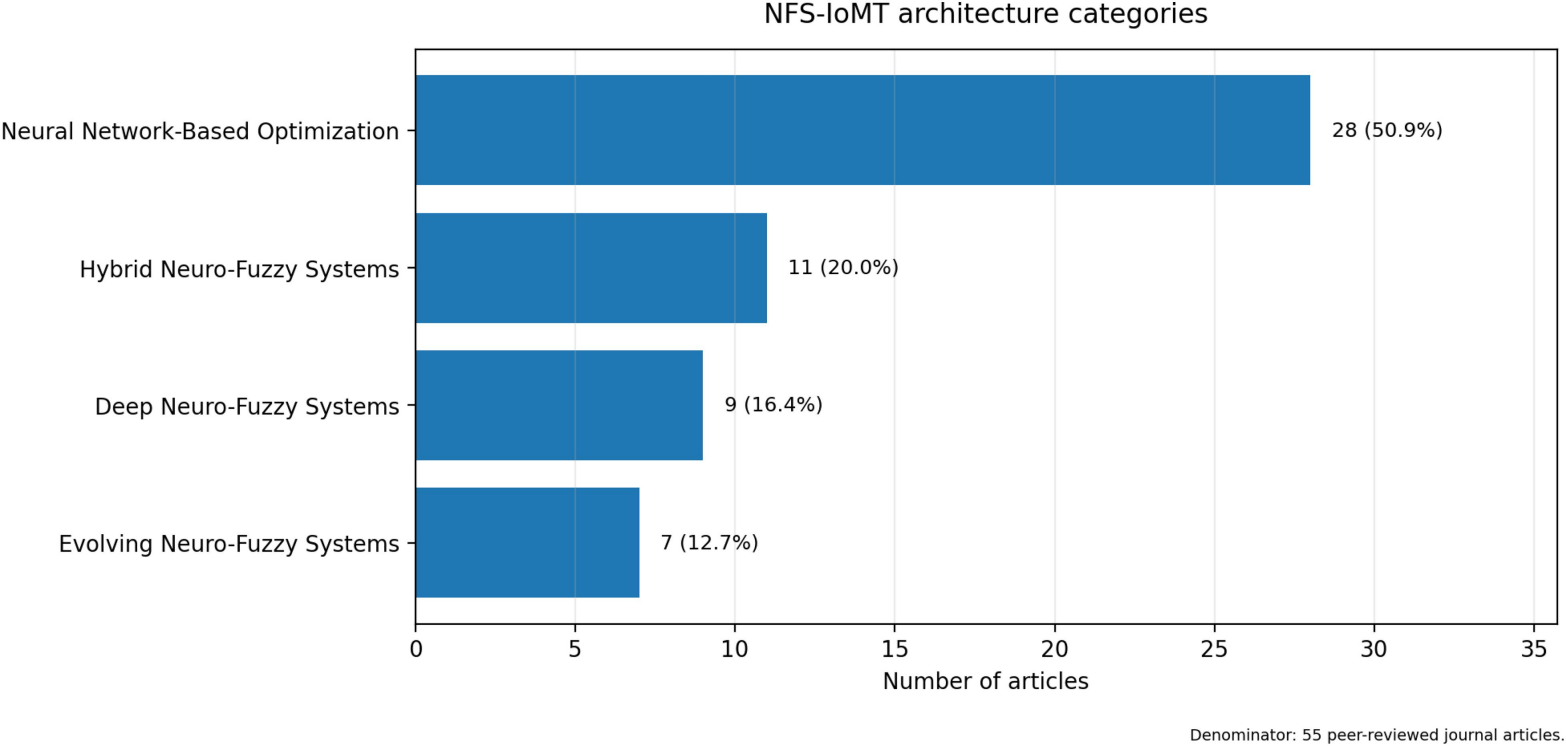
Distribution of primary neuro-fuzzy system architecture categories across the 55 peer-reviewed journal articles included in the systematic evidence base.

Predictive neuro-fuzzy systems have also been reported for Parkinson’s disease estimation or diagnosis (32, 35), kidney disease prediction (61), autism screening (37), stress prediction (34), generalized disease prediction (41), multi-disease severity prediction (44, 57, 70, 76), anxiety prediction (47), hydrotherapy safety and body-temperature prediction (33), ischemic heart disease therapy prediction (62), and electroencephalography-based neuro-monitoring (78). Figure 4 reports the corresponding primary medical-domain distribution.

Overall, predictive systems represent the largest branch of the application-domain taxonomy. Many included peer-reviewed journal articles report accuracy, precision, sensitivity or recall, specificity, F1-score, coefficient of determination, mean squared error, or root mean squared error. However, most included peer-reviewed journal articles remain model-performance oriented and provide limited evidence on deployment-sensitive metrics such as bandwidth use, end-to-end latency, energy consumption, memory footprint, and robustness under continuous streaming conditions.

#### 6.2.2 Detection systems

Detection systems identify disease presence, abnormal physiological states, or clinically significant events. In IoMT contexts, this includes arrhythmia recognition, stress detection, abnormal electroencephalography pattern identification, heart sound analysis, and image-based disease detection. Neuro-fuzzy systems are relevant to such tasks because they can tolerate uncertainty and noise while preserving rule-based decision structures that can be evaluated for interpretability (15).

The included detection-oriented articles cover cardiovascular, oncological, neurological, affective-health, and medical-image contexts. Cardiovascular and cardiac-audio detection include heart sound disorder detection (80), heart murmur identification (84), pulse-pattern diagnosis (30), arrhythmia detection (73), and cardiovascular diagnosis from heart sounds or multimodal inputs (63). Oncology-related detection includes acute leukemia classification (60), breast cancer diagnosis (49), lung cancer detection (42), osteosarcoma detection (71), brain tumour detection (31, 54), and cervical cancer diagnosis (83).

Detection-oriented neuro-fuzzy systems have also been applied to Parkinson’s freezing-of-gait detection (52), schizophrenia detection from electroencephalography signals (36), wearable stress detection (74, 82), and electroencephalography-based emotion recognition in centralized or federated settings (75). Several included articles report strong classification results. However, as with predictive systems, deployment-aware evaluation remains limited. Many included articles do not report latency, throughput, computational cost, energy consumption, memory footprint, or communication reliability, even where the application implies real-time or distributed operation.

#### 6.2.3 Care-support systems

Care-support systems assist clinicians, caregivers, and patients in the ongoing management of health conditions, particularly in remote monitoring, chronic care, and rehabilitation settings. In such applications, the role of neuro-fuzzy systems extends beyond classification toward continuous assistance, patient-state interpretation, and decision support. Despite this clinical importance, only 1 of the 55 included peer-reviewed journal articles (1.8%) was classified primarily as a care-support system.

The limited evidence identified in this branch suggests that neuro-fuzzy systems may support heterogeneous data integration and remote decision support, but the field remains underdeveloped relative to prediction and detection. The included care-support evidence does not provide sufficient evaluation of latency, communication efficiency, energy consumption, memory use, scalability, or integration with real-world clinical workflows. This indicates a clear opportunity for future research on deployment-aware neuro-fuzzy care-support systems for continuous and resource-constrained healthcare environments.

#### 6.2.4 Recommender systems

Recommender systems refer to neuro-fuzzy systems that propose diagnostic actions, therapeutic strategies, or personalized treatment options based on patient profiles and clinical data. The neuro-fuzzy formulation is attractive in this setting because it combines data-driven adaptation with rule-based reasoning that should be evaluated for clinical readability, fidelity, and auditability. However, only 1 of the 55 included peer-reviewed journal articles (1.8%) was classified primarily as a recommender system.

The limited evidence base shows that recommender applications remain at an early stage in IoMT-enabled neuro-fuzzy research. Existing work illustrates the potential of neuro-fuzzy reasoning for personalized decision support, but broader generalization is constrained by the lack of live IoMT integration, real-time evaluation, latency analysis, operational validation, and prospective clinical assessment. Future recommender systems should therefore move beyond isolated predictive performance and examine whether neuro-fuzzy recommendations can operate reliably and safely within connected clinical workflows.

Taken together, the application-domain analysis shows that neuro-fuzzy systems have been applied across a broad range of healthcare functions, but the distribution is highly uneven. Predictive systems dominate the included evidence base, followed by detection systems, while care-support and recommender systems remain rare. Across all categories, performance is usually reported using model-level metrics such as accuracy, sensitivity or recall, specificity, precision, F1-score, root mean squared error, area under the curve, or coefficient of determination. However, system-level measures such as latency, energy consumption, bandwidth use, memory footprint, and deployment robustness are reported inconsistently or not at all. This limits the ability to assess practical readiness for real-world IoMT deployment.

### 6.3 Neuro-fuzzy system deployment environment in internet of medical things

The deployment environment of a neuro-fuzzy system is a critical determinant of responsiveness, scalability, privacy, energy demand, and suitability for real-world IoMT applications. In the included evidence base, deployment environments were grouped as cloud or cloud-based, fog-cloud or fog-oriented, edge-cloud, and not clearly specified.

The article-level evidence indicates that deployment reporting remains limited. Thirty-four of the 55 included peer-reviewed journal articles (61.8%) did not clearly specify the deployment layer in the submitted manuscript or extraction table. Among the articles that did report a deployment setting, 15 articles (27.3%) used cloud or cloud-based execution, 5 articles (9.1%) used fog-cloud or fog-oriented execution, and 1 article (1.8%) used an edge-cloud setting. These results show that cloud-centric and semi-centralized deployment remains more common than explicit edge-based execution, while a majority of included peer-reviewed journal articles do not provide enough information to determine the deployment layer with confidence.

Cloud-based deployments are used because they provide centralized storage, scalable computing resources, and flexibility for model training and inference. This pattern is visible in cloud-Internet of Things or cloud-Internet of Medical Things articles on multi-disease prediction, heart monitoring, disease risk prediction, secure diagnosis, and medical image analysis (58, 59, 66, 69, 70, 72, 76). These included articles often report strong predictive or diagnostic performance, but many do not evaluate communication delay, network utilization, bandwidth cost, energy consumption, or memory use.

Fog-cloud and fog-oriented deployments are less common but more closely aligned with latency-sensitive healthcare applications. Fog-related articles include cognitive-health monitoring (47), Parkinson’s or heart disease-related prediction (35, 48), diabetes prediction (51), and blockchain-enabled fog-cloud diagnostic support (46). These included articles suggest that fog computing may support more responsive decision-making, but most remain simulation-based or benchmark-oriented and provide limited evidence on sustained throughput, memory footprint, energy use, and long-term performance under realistic network conditions.

Edge-cloud deployment is the least represented category in the coded evidence base, with only 1 article (1.8%) classified as edge-cloud. This is notable because edge execution is particularly important for time-sensitive, privacy-preserving, and low-connectivity IoMT use cases. Some included articles conceptually align with edge or wearable deployment, especially those involving wearable stress detection, biofeedback monitoring, or real-time sensing (34, 77, 81, 82), but the submitted manuscript and extraction table do not consistently provide enough operational evidence to classify them as explicit edge deployments.

Deployment-sensitive evaluation was also limited. Quantitative latency or processing-time evidence was reported in only 6 included peer-reviewed journal articles (10.9%). A further 8 articles (14.5%) mentioned latency or low-latency relevance without reporting a quantitative latency value. The remaining 41 articles (74.5%) did not report latency in the submitted manuscript or extraction table. Energy-consumption reporting and memory or computational-resource reporting were not available for any of the 55 included peer-reviewed journal articles in the extracted evidence base. This finding is important because IoMT systems often operate under strict latency, power, bandwidth, and resource constraints.

Overall, the deployment analysis reveals a persistent mismatch between the clinical requirements of IoMT systems and the evaluation practices of much of the neuro-fuzzy literature. Cloud-centric implementations remain more visible than fog or edge-cloud deployments, and most included peer-reviewed journal articles do not clearly specify execution context. Across all deployment categories, system-level measures such as latency, energy consumption, memory footprint, bandwidth use, throughput, and network robustness are rarely reported. Consequently, although neuro-fuzzy systems show promise for connected healthcare, much of the evidence remains grounded in model-level performance rather than operationally realistic deployment analysis. Advancing the field will require standardized deployment-aware evaluation frameworks that assess predictive performance together with end-to-end system behaviour under practical IoMT constraints.

## 7 Analysis of results

Building on the methodology described in Section 5, this section presents the results of the systematic literature review. The analysis is organized around the review questions and the proposed taxonomy, with emphasis on neuro-fuzzy system architecture, healthcare application domain, deployment environment, and evaluation practice. Unless otherwise stated, all reported percentages use the denominator of 55 included peer-reviewed journal articles.

To improve traceability, the article-level evidence supporting the synthesis is provided in Supplementary Table S1. For each of the 55 included peer-reviewed journal articles, the table reports the author and year, disease or application area, data source, dataset type, neuro-fuzzy system type, static or evolving classification, deployment layer, evaluation metrics, latency, energy, or memory reporting, and main limitation. This supplementary table provides the article-level basis for the aggregate architectural, application-domain, deployment, and evaluation findings reported in the main text.

### 7.1 Neuro-fuzzy system architectures in IoMT

**Figure 5:**
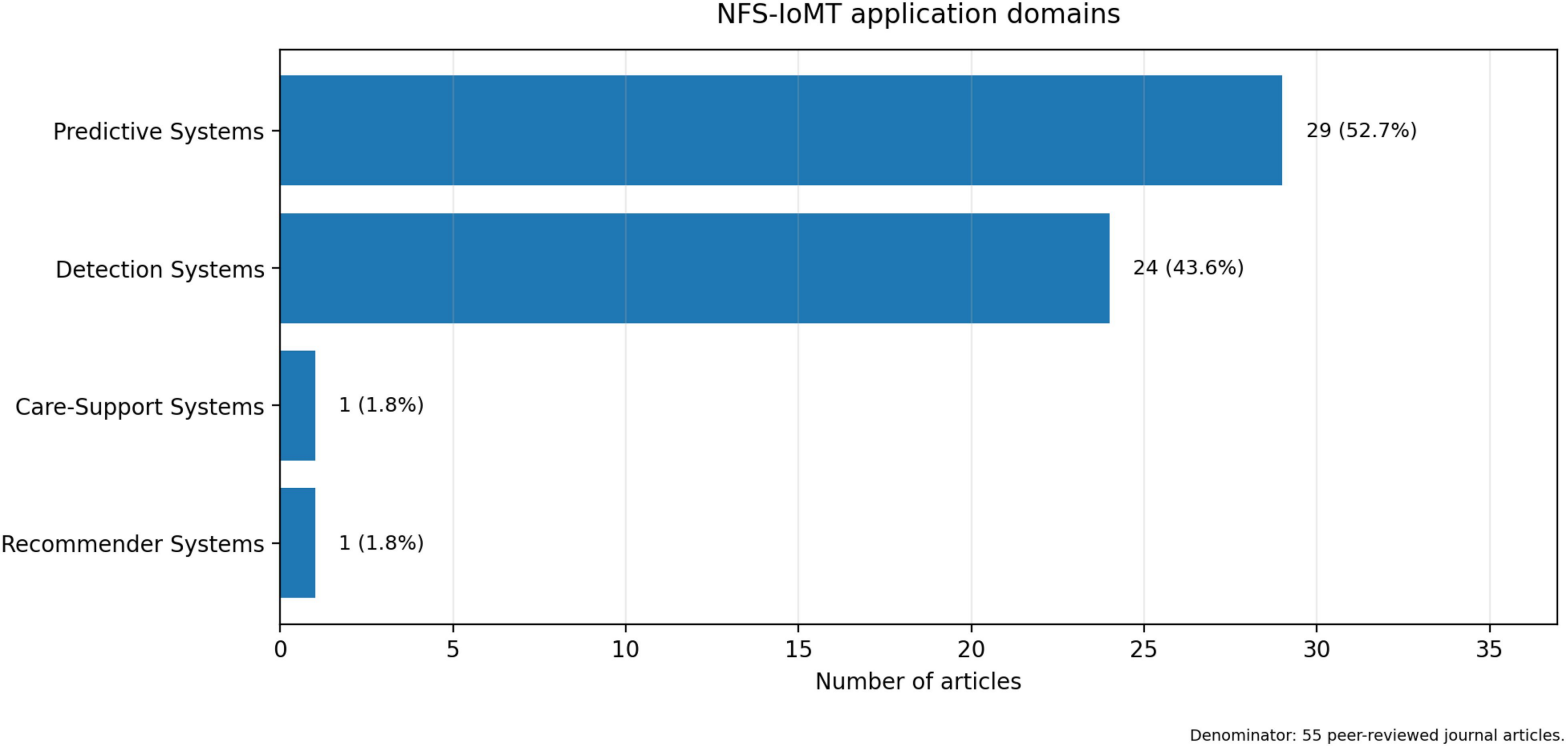
Distribution of primary neuro-fuzzy system application domains across the 55 peer-reviewed journal articles included in the systematic evidence base.

The architecture of a neuro-fuzzy system plays a central role in determining its suitability for deployment in real-time, resource-constrained IoMT environments. As healthcare systems move toward connected monitoring through wearables, implantable devices, mobile platforms, and distributed sensing infrastructures, the underlying models must support not only predictive performance but also adaptability, interpretability, and operational feasibility. This subsection addresses **RQ3**: *How are NFS architectures for IoMT taxonomically classified across deployment environments and functional roles?* Figure 5 summarizes the resulting primary architecture distribution.

The revised evidence base consists of **55 peer-reviewed journal articles**. Based on the article-level coding, static neuro-fuzzy systems dominate the included evidence base, accounting for **48 articles (87.3%)**, while evolving neuro-fuzzy systems account for **7 articles (12.7%)**. At the architectural-category level, **Neural-Network-Based Optimization** represents the largest group, comprising **28 articles (50.9%)**. These systems integrate fuzzy inference with neural learning, evolutionary strategies, or metaheuristic optimization to tune membership functions, rule bases, feature-selection procedures, or consequent parameters (30–57).

**Hybrid Neuro-Fuzzy Systems** account for **11 articles (20.0%)** (67–77). These systems combine fuzzy inference with complementary computational methods such as deep learning, recurrent neural networks, ensemble learning, regression trees, federated learning, and metaheuristic optimization.

**Deep Neuro-Fuzzy Systems** account for **9 articles (16.4%)** (58–66). These systems embed fuzzy reasoning within deep learning pipelines, typically using convolutional or recurrent architectures for high-dimensional medical images, multimodal signals, or physiological time-series data.

**Evolving Neuro-Fuzzy Systems** account for **7 articles (12.7%)** (78–84). These systems introduce online learning, structural plasticity, incremental rule adaptation, and single-pass learning. They are conceptually well suited to adaptive IoMT environments because they can respond to patient variability, changing sensor patterns, and non-stationary data streams, although they remain underrepresented and insufficiently validated in real-world deployment settings.

Taken together, the architectural evidence shows that the field remains dominated by static, accuracy-oriented systems. Neural-network-based optimization and hybrid systems together account for **39 articles (70.9%)**, indicating that the included evidence base still prioritizes training-time optimization and model fusion. Deep neuro-fuzzy systems show relevance for high-dimensional and multimodal healthcare data, while evolving neuro-fuzzy systems remain comparatively rare despite their stronger conceptual fit with streaming and adaptive IoMT environments. Across all architectural groups, limited reporting of latency, energy consumption, memory footprint, bandwidth use, and sustained runtime performance constrains the assessment of practical readiness. Future research should therefore prioritize deployment-aware benchmarks, hardware-in-the-loop evaluation, continual-learning stability, and interpretability evaluation for adaptive rules under realistic edge, fog, and cloud conditions.

The next subsection extends this analysis from architecture to clinical functionality by examining how neuro-fuzzy systems are applied across prediction, detection, care-support, and recommender roles within the IoMT ecosystem.

### 7.2 NFS IoMT application domain

This subsection addresses **RQ1**, **RQ2**, and **RQ4** by analysing how neuro-fuzzy systems are used across clinical functions, what motivates their adoption, and how their performance is evaluated. The discussion follows the taxonomy presented in Figure 3, which classifies application domains into *Prediction Systems*, *Detection Systems*, *Care-Support Systems*, and *Recommender Systems*.

**Figure 6:**
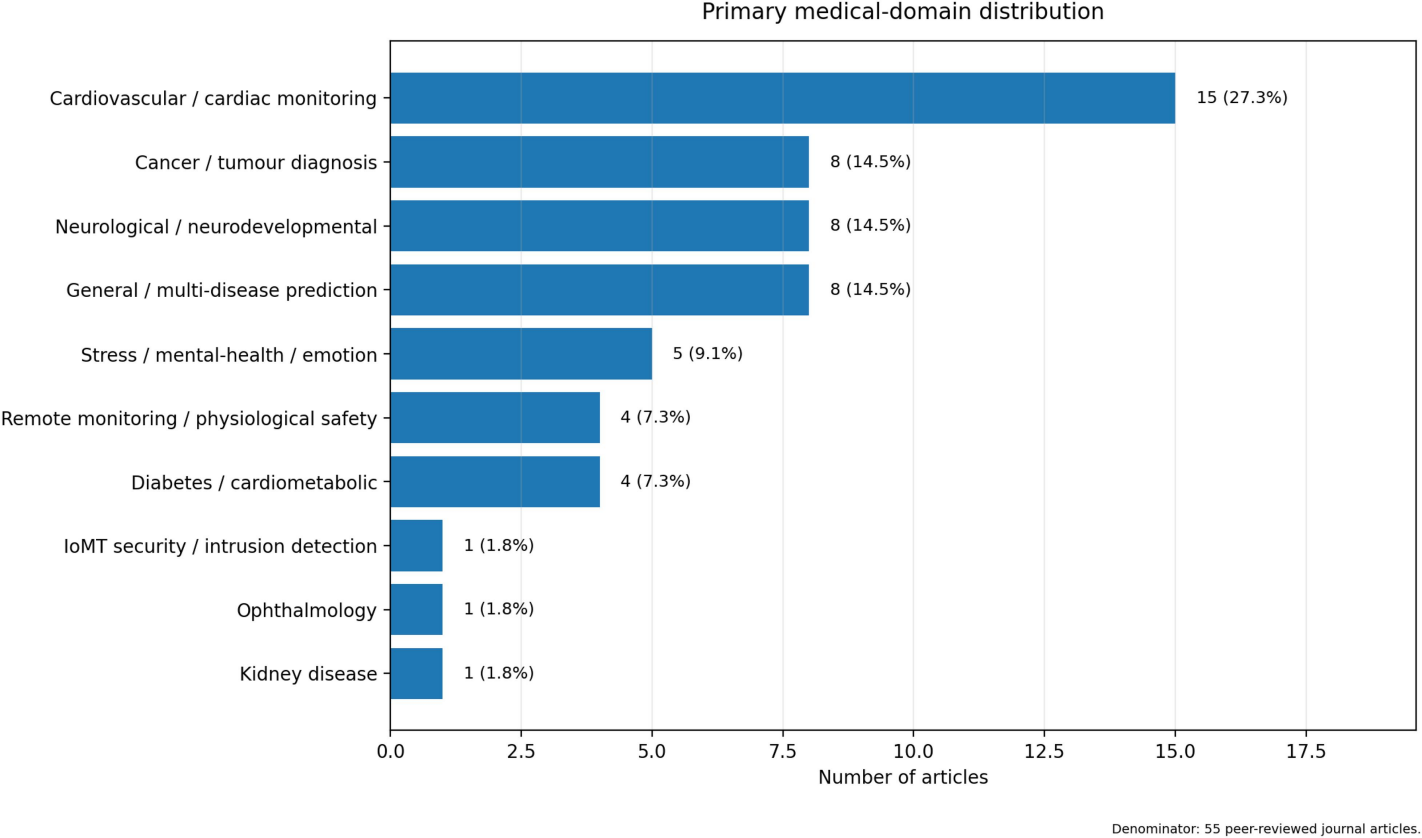
Distribution of primary medical domains across the 55 peer-reviewed journal articles included in the systematic evidence base.

The revised systematic evidence base of 55 peer-reviewed journal articles shows a strong concentration in prediction and detection tasks. **Prediction Systems** account for **29 articles (52.7%)**, while **Detection Systems** account for **24 articles (43.6%)**. By contrast, **Care-Support Systems** and **Recommender Systems** account for only **1 article each (1.8% each)**. This distribution confirms that current neuro-fuzzy research in IoMT is heavily focused on disease prediction, diagnostic classification, and abnormality detection, while longitudinal care support and personalized recommendation remain weakly represented. The application-domain distribution is shown in Figure 6.

**Prediction Systems** represent the largest application group. These systems are used to anticipate disease risk, disease progression, patient status, or adverse clinical events before they occur. The included predictive articles are concentrated mainly in cardiovascular, metabolic, neurological, mental-health, and multi-disease prediction contexts. Cardiovascular applications include heart disease diagnosis, wearable heart monitoring, and broader cardiovascular risk assessment (38, 43, 48, 50, 66, 67, 69, 72, 77). Diabetes prediction is another major cluster, with cloud, fog-cloud, and benchmark-based Adaptive Neuro-Fuzzy Inference System formulations (39, 46, 51, 56). Additional predictive applications include Parkinson’s disease estimation or diagnosis (32, 35), kidney disease prediction (61), autism screening (37), stress prediction (34), generalized disease prediction (41), multi-disease severity prediction (44, 57, 70, 76), anxiety prediction (47), hydrotherapy safety and body-temperature prediction (33), ischemic heart disease therapy prediction (62), and electroencephalography-based neuro-monitoring (78).

The included predictive articles commonly use benchmark datasets such as UCI, PhysioNet, MIT-BIH, PTB, Kaggle-derived datasets, hospital records, electronic health records, wearable-sensor data, and condition-specific clinical datasets. Reported evaluation metrics include accuracy, sensitivity or recall, specificity, precision, F1-score, root mean squared error, mean squared error, area under the curve, and coefficient of determination. Although many included articles report model-level performance, deployment-oriented metrics remain limited. Quantitative latency or processing-time evidence is reported in only a small subset of included articles, and most predictive articles do not report energy consumption, memory footprint, bandwidth use, or sustained runtime performance.

**Detection Systems** constitute the second largest application group, accounting for **24 articles (43.6%)**. These systems focus on identifying disease presence, abnormal physiological states, or clinically significant events. Common detection contexts include arrhythmia recognition (73), cardiovascular diagnosis from heart sounds or multimodal inputs (63, 80, 84), pulse-pattern diagnosis (30), stress identification (74, 82), and electroencephalography-based emotion or mental-state recognition (36, 75). Detection articles also address acute leukemia (60, 68), breast cancer (49), lung cancer (42), osteosarcoma (71), brain tumour detection (31, 54), and cervical cancer diagnosis (83).

Detection systems often report diagnostic or classification performance using conventional model-level metrics. However, the practical demands of real-time IoMT detection are not adequately reflected in most evaluations. Few included peer-reviewed journal articles examine packet loss, jitter, throughput, end-to-end latency, energy consumption, communication cost, or runtime behaviour under constrained edge or fog conditions. This creates a gap between diagnostic performance and operational readiness.

**Figure 7:**
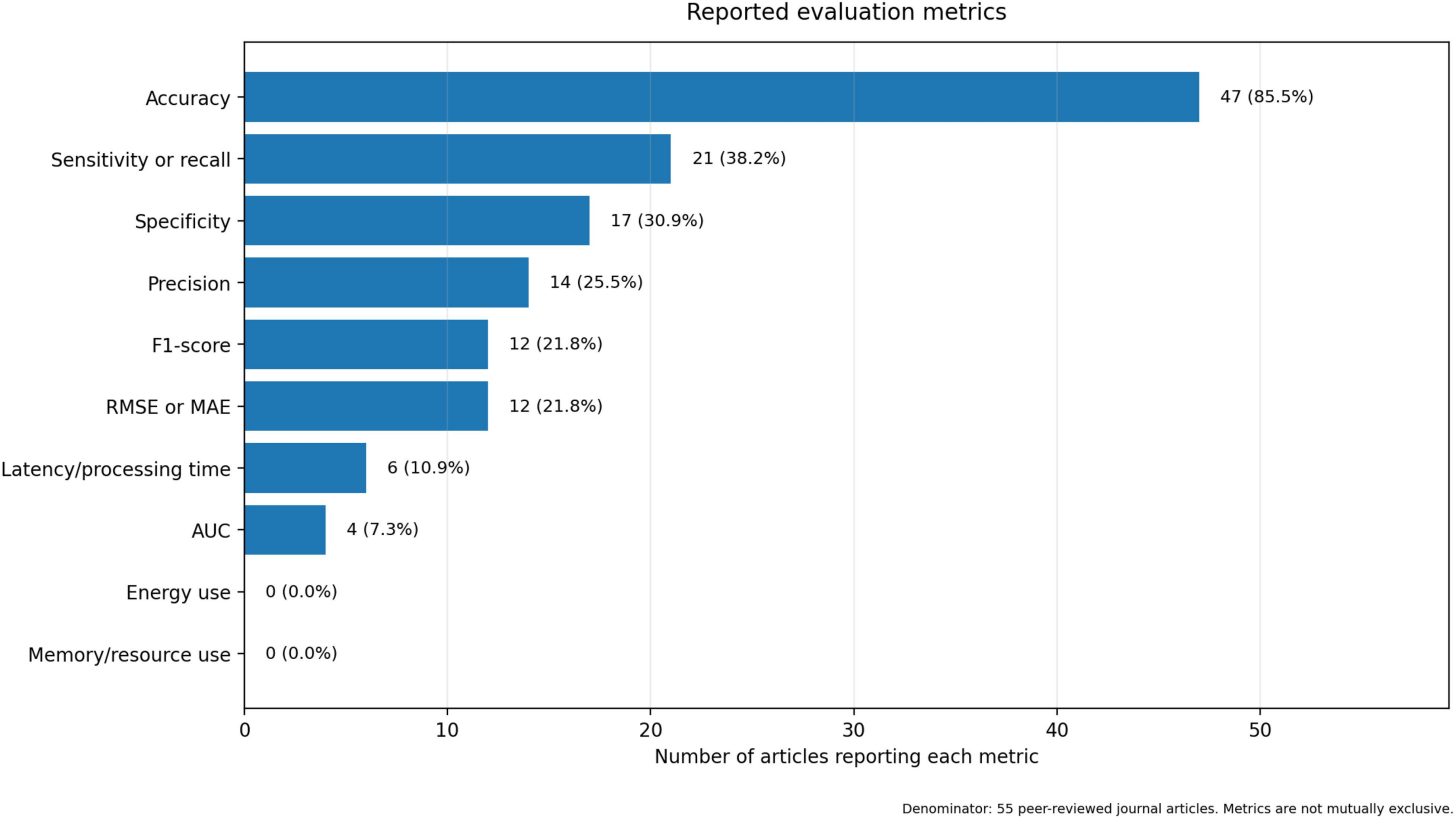
Prevalence of performance evaluation metrics reported by the 55 peer-reviewed journal articles included in the systematic evidence base.

**Care-Support Systems** and **Recommender Systems** are the least represented application categories, with **1 article each (1.8% each)**. Care-support systems are intended to assist clinicians, caregivers, or patients in continuous monitoring, chronic disease management, rehabilitation, and remote clinical assistance. Recommender systems are intended to provide personalized treatment, diagnostic, or lifestyle recommendations based on patient profiles and clinical data. The limited number of included peer-reviewed journal articles in these categories shows that clinically important functions such as longitudinal care support, therapy guidance, and personalized decision support remain underdeveloped in the current NFS-IoMT evidence base. Figure 7 summarizes the frequency of reported evaluation metrics across the included articles.

Across all application domains, the main motivation for using neuro-fuzzy systems is their ability to combine data-driven learning with rule-based fuzzy reasoning that can be evaluated for interpretability under uncertainty. This is valuable in IoMT contexts where data may be noisy, heterogeneous, incomplete, or patient-specific. Nevertheless, the evidence shows a persistent imbalance between model-level and system-level evaluation. In the extracted evidence base, accuracy was reported in 47 articles, sensitivity or recall in 21 articles, specificity in 17 articles, precision in 14 articles, F1-score in 12 articles, root mean squared error or mean absolute error in 12 articles, and area under the curve in 4 articles. By contrast, quantitative latency or processing-time evidence was reported in only 6 articles, while energy consumption and memory or computational-resource reporting were absent from the extracted evidence base. As a result, the included evidence base indicates potential for neuro-fuzzy systems in prediction and detection, but provides limited evidence of practical or clinical readiness for continuous, resource-constrained, and clinically integrated IoMT deployment.

### 7.3 NFS-IoMT deployment environment

**Figure 8:**
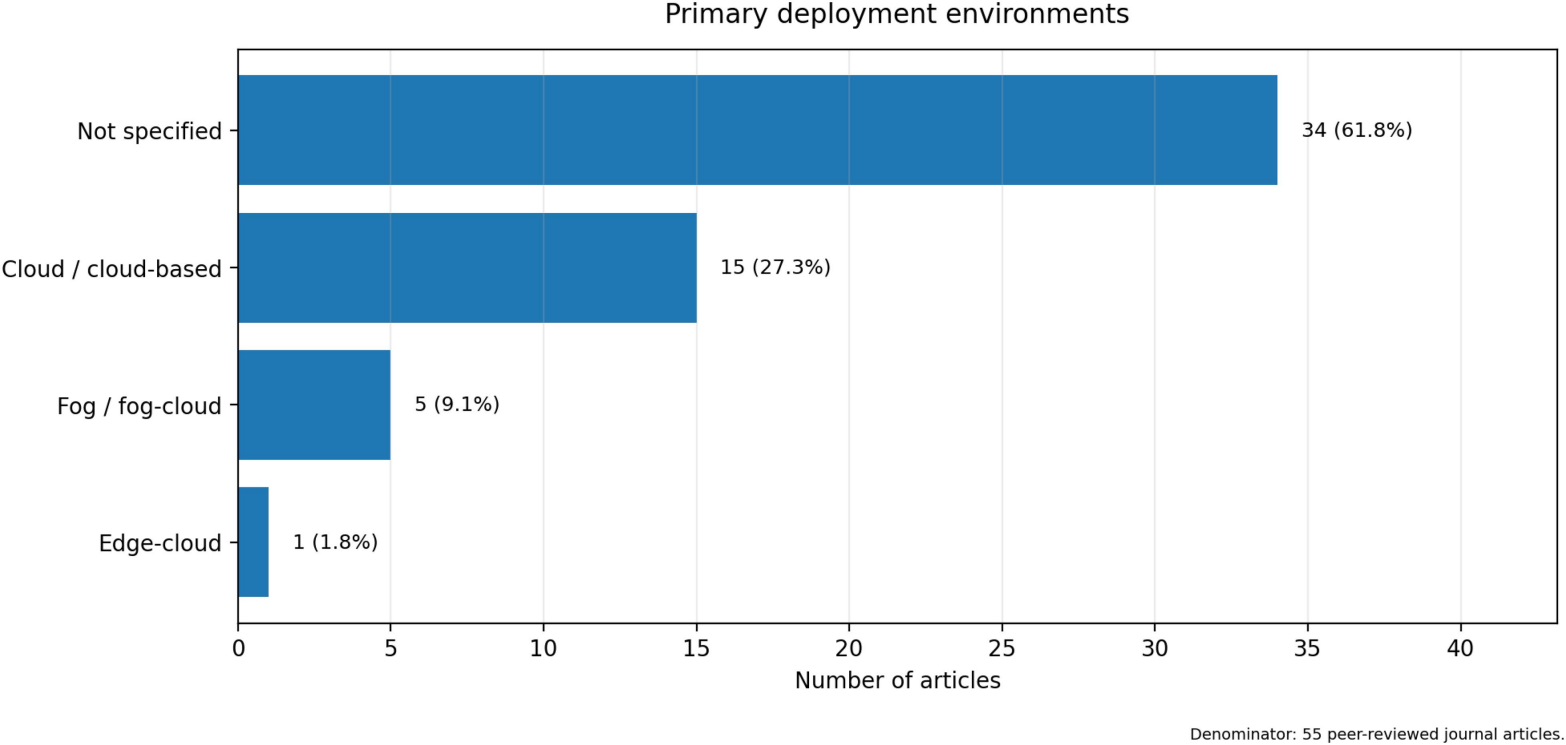
Distribution of primary NFS-IoMT deployment environments across the 55 peer-reviewed journal articles included in the systematic evidence base.

The deployment environment of a neuro-fuzzy system is a decisive factor in determining responsiveness, scalability, privacy, energy demand, and suitability for real-world IoMT applications. The taxonomy distinguishes between cloud, fog-cloud, edge-cloud, and unspecified deployment environments, but the revised evidence base shows that deployment reporting remains incomplete across many included peer-reviewed journal articles. Figure 8 summarizes the primary deployment-environment distribution.

Of the **55 included peer-reviewed journal articles**, **34 articles (61.8%)** did not clearly specify the deployment layer in the submitted manuscript or extraction table. Among articles that did report a deployment context, **15 articles (27.3%)** used cloud or cloud-based Internet of Things or Internet of Medical Things settings, **5 articles (9.1%)** used fog-cloud or fog-oriented settings, and **1 article (1.8%)** used an edge-cloud setting. These results show that the included evidence base remains dominated by unspecified or cloud-centric execution, while explicit fog and edge-cloud deployment remain limited.

Cloud environments are commonly used because they provide centralized storage, computational scalability, and support for large-scale model training and inference. Cloud-based articles include multi-disease prediction, cloud-IoT heart monitoring, disease risk prediction, secure diagnosis, and medical image analysis (58, 59, 66, 69, 70, 72, 76). These included articles often report predictive or diagnostic performance, but many do not evaluate communication delay, network utilization, bandwidth cost, energy consumption, or memory use.

Fog-cloud and fog-oriented deployments are less common but more closely aligned with latency-sensitive healthcare applications. Fog-related articles include cognitive-health monitoring (47), Parkinson’s or heart-disease-related prediction (35, 48), diabetes prediction (51), and blockchain-enabled fog-cloud diagnostic support (46). These included articles suggest that fog computing may support more responsive decision-making, but most remain simulation-based or benchmark-oriented and provide limited evidence on sustained throughput, memory footprint, energy use, and long-term performance under realistic network conditions.

Edge-cloud deployment is the least represented category in the coded evidence base, with only **1 article (1.8%)** classified as edge-cloud. This is notable because edge execution is especially important for time-sensitive, privacy-preserving, and low-connectivity IoMT use cases. Some included articles conceptually align with edge or wearable deployment, particularly those involving wearable stress detection, biofeedback monitoring, or real-time sensing (34, 77, 81, 82), but the available extracted evidence does not consistently provide enough operational detail to classify them as explicit edge deployments.

Deployment-sensitive evaluation is also limited. Quantitative latency or processing-time evidence is reported in only **6 included peer-reviewed journal articles (10.9%)**. A further **8 articles (14.5%)** mention latency, low-latency relevance, or processing efficiency without reporting a quantitative latency value. The remaining **41 articles (74.5%)** do not report latency in the submitted manuscript or extraction table. Energy-consumption reporting and memory or computational-resource reporting are not available for any of the 55 included peer-reviewed journal articles in the extracted evidence base.

Overall, the deployment analysis reveals a persistent mismatch between the clinical requirements of IoMT systems and the evaluation practices of much of the neuro-fuzzy literature. Cloud-centric and unspecified deployments remain dominant, while fog and edge-cloud deployments are comparatively underexplored. Across all deployment categories, system-level measures such as latency, energy consumption, memory footprint, bandwidth use, throughput, and network robustness are rarely reported. Consequently, although neuro-fuzzy systems show promise for connected healthcare, much of the evidence remains grounded in model-level performance rather than operationally realistic deployment analysis. Future studies should therefore adopt deployment-aware evaluation frameworks that assess predictive performance together with end-to-end system behaviour under practical IoMT constraints.

## 8 Challenges, open issues, and future research directions

Despite strong model-level performance, the included evidence base remains at an early stage of technological and clinical maturity. Most systems are retrospective, experimental, benchmark-driven, simulation-based, or prototype-level, and many are evaluated under controlled conditions rather than live care settings. Evidence of prospective validation, safety assessment, clinician-usability testing, patient acceptability assessment, regulatory planning, and integration with electronic health record or telemedicine workflows remains limited (90, 91). This section addresses **RQ5** by summarizing the main technical, operational, and translational barriers affecting NFS in IoMT.

### 8.1 Static architectures and limited adaptive learning

A major limitation is the dominance of static NFS architectures. Static models are trained offline and usually retain fixed structures and parameters during inference. This is problematic in IoMT because patient physiology, sensor quality, device calibration, and clinical context may change over time. Static systems may therefore degrade under concept drift unless they are retrained or recalibrated (18). Evolving NFS provide a stronger conceptual fit because they support online learning, rule adaptation, and structural plasticity (78, 82, 84). However, current evolving systems remain underrepresented and are often tested using narrow datasets or offline streams. Important issues such as rule growth, update stability, memory overhead, catastrophic forgetting, and long-term performance drift require deeper evaluation. Future models should incorporate bounded adaptation, pruning, novelty detection, uncertainty-aware updating, and resource-aware learning.

### 8.2 Deployment-aware and real-world validation

The evidence base shows a persistent gap between algorithmic accuracy and operational validation. Many included peer-reviewed journal articles report high accuracy, sensitivity, specificity, or F1-score, but few evaluate behaviour in live IoMT settings, multi-site cohorts, clinician-in-the-loop workflows, or realistic network conditions. Guidance for clinical AI reporting and early-stage evaluation stresses the need to describe the intended use setting, human-AI interaction, inputs and outputs, error handling, safety considerations, and validation design rather than relying only on retrospective model performance (90–93). High retrospective performance does not establish deployment readiness when latency, throughput, missing data, packet loss, device instability, energy consumption, and workflow constraints are not assessed. Future work should include real-time simulation, hardware-in-the-loop testing, edge/fog/cloud testbeds, digital twins, and controlled clinical pilots. Studies claiming real-time or distributed IoMT relevance should report inference latency, update latency, memory use, energy demand, communication overhead, throughput, robustness, failure recovery, and the clinical workflow in which outputs would be reviewed or acted on.

### 8.3 Cloud, fog, edge, and interoperability constraints

The deployment layer strongly affects feasibility. Cloud computing offers scalable storage and computation but can introduce latency, bandwidth dependency, and privacy concerns. Fog computing can reduce round-trip delay by processing data closer to sensors, but task offloading, synchronization, node failure, quality of service, and security remain underreported. Edge execution is essential for wearable, mobile, low-connectivity, and privacy-sensitive monitoring, but edge nodes are constrained by memory, processing power, and battery capacity. These trade-offs are consistent with broader IoT and connected-health infrastructure work, where computation placement affects delay, bandwidth use, privacy exposure, and energy demand (94–96). Future NFS-IoMT systems should therefore use lightweight inference engines, compressed rule bases, quantized parameters, adaptive partitioning, and energy-aware updates.

Interoperability is equally important. Many reviewed systems are isolated analytical models rather than components of connected healthcare infrastructure. Future systems should support secure streaming pipelines, modular application programming interfaces, identity and access management, audit trails, and compatibility with healthcare and IoT standards such as HL7 FHIR for health data exchange, DICOM for medical imaging information, ISO/IEEE 11,073 for personal health-device communication, MQTT for lightweight publish/subscribe messaging, and CoAP for constrained IoT communication (97–102). These standards and protocols can support data exchange, device communication, and application integration across electronic health record, telemedicine, and remote-monitoring workflows.

### 8.4 Interpretability, auditability, and clinical translation

NFS are often described as interpretable, but formal fuzzy-rule structure should be treated as a starting point for evaluation rather than proof of practical clinical interpretability. Practical clinical interpretability must be demonstrated through rules that are concise, clinically meaningful, stable, faithful to model behaviour, and understandable in real workflows (103–106). Deep, hybrid, ensemble, and evolving NFS can reduce interpretability through latent representations, large rule bases, overlapping rules, and changing structures. Rule-base size, rule length, membership-function structure, rule overlap, rule stability, clinician readability, explanation fidelity, and auditability should therefore be reported as evaluation requirements rather than assumed properties of the model. Evolving systems should also report rule-growth control, update frequency, drift response, pruning criteria, version history, and audit logs.

Clinical translation requires more than formal fuzzy rules and high retrospective accuracy. Prospective validation, external multi-site testing, usability assessment, safety evaluation, workflow analysis, and regulatory planning are necessary before routine clinical use can be claimed. Adaptive systems also require safe update policies, version control, rollback mechanisms, monitoring thresholds, and human approval for clinically significant rule changes. Clinician trust should be assessed empirically because explanations can support appropriate reliance only when they are clear, truthful, clinically relevant, and aligned with user needs (107).

### 8.5 Privacy, security, reproducibility, and reporting standards

IoMT systems process sensitive health data across devices, networks, and institutions. Privacy and security should therefore be integrated into sensing, transmission, inference, model updating, storage, and reporting. Federated learning and privacy-preserving analytics are promising because they can support multi-site learning without centralizing raw patient data, but they introduce challenges involving communication overhead, non-independent and non-identically distributed data, model synchronization, rule aggregation, and cross-site explainability (75). Blockchain or distributed ledgers may strengthen auditability but can also introduce latency, storage, and governance costs.

Reproducibility is limited by variation in datasets, splits, baselines, validation strategies, deployment assumptions, and reporting practices. Future studies should report dataset source and size, clinical task, input modality, preprocessing, validation strategy, baselines, model complexity, interpretability-evaluation outputs, calibration, deployment environment, latency, memory use, energy use, communication overhead, privacy safeguards, and limitations. Table 7 summarizes a minimum reporting framework that separates model-level metrics, system-level deployment metrics, and clinical-translation metrics. The metric groups align with common prediction-model reporting practice and early-stage clinical AI evaluation guidance, while remaining adapted to NFS-IoMT studies (90, 91).

**Table 7:** Minimum reporting framework for future neuro-fuzzy systems in IoMT evaluation, separated into model-level, system-level deployment, and clinical-translation metrics.

Reporting layer	Evaluation item	Minimum information to report	Common measures or indicators	Purpose for NFS-IoMT evaluation
Model-level metrics	Predictive performance and validation	Dataset source and size, clinical task, data split, internal validation, external validation where available, class-imbalance handling, baselines, and confidence intervals where appropriate	Confusion matrix, accuracy, balanced accuracy, sensitivity or recall, specificity, precision or PPV, NPV, F1-score, ROC-AUC, PR-AUC, MAE, RMSE	Shows whether the model performs adequately and whether results are comparable with relevant alternatives
Model-level metrics	Calibration, uncertainty, and clinical utility	Reliability of probability or risk estimates, uncertainty handling, calibration method, and decision-threshold rationale	Calibration plot, calibration-in-the-large, calibration slope, Brier score, expected calibration error, uncertainty intervals, decision curve, net benefit	Distinguishes discrimination from reliable risk estimation and supports cautious interpretation of clinical decision support claims
Model-level metrics	Interpretability evaluation and model complexity	Rule-base properties, membership-function structure, explanation format, model size, adaptation behaviour, clinician readability, explanation fidelity, and auditability of evolving updates	Number of rules, rule length, rule-base size, membership functions, rule overlap, rule stability, clinician readability rating, explanation fidelity, update frequency, version history, pruning logs, audit logs	Tests whether interpretability is demonstrated, traceable, and manageable in connected-health settings rather than assumed from the use of fuzzy rules
System-level deployment metrics	Runtime behaviour and throughput	Execution environment, hardware or simulator details, inference timing, update timing, stream rate, and deadline assumptions	Inference latency, end-to-end latency, update latency, samples per second, throughput, jitter, deadline-miss rate, patients monitored per gateway	Shows whether the system can support real-time or streaming IoMT operation
System-level deployment metrics	Resource use and communication overhead	Compute, memory, energy, model-size, and network-demand measurements under stated edge, fog, cloud, or hybrid conditions	CPU or GPU use, RAM use, model size, number of parameters or rules, energy per inference, battery impact, bytes transmitted, bandwidth use, communication rounds, packet loss	Assesses suitability for wearable, edge, fog, cloud, and low-connectivity healthcare environments
System-level deployment metrics	Robustness, resilience, interoperability, security, and privacy	Behaviour under noisy signals, missing data, sensor dropout, drift, connectivity failures, failover, data exchange, access control, and privacy-preserving operation	Missingness tests, noise tests, drift scenarios, uptime, failover rate, recovery time, HL7 FHIR, DICOM, IEEE 11073, MQTT, CoAP, encryption, authentication, federated learning, audit logs	Shows whether the system can operate safely and reliably within connected healthcare infrastructure
Clinical-translation metrics	Clinical validation and generalizability	External validation, prospective testing, multi-site evaluation, subgroup performance, and relevance to clinical endpoints	External validation, prospective pilot, multi-site cohort, subgroup or fairness analysis, clinical endpoint, decision curve, net benefit	Separates algorithmic promise from evidence that may support future clinical translation
Clinical-translation metrics	Human factors and workflow fit	Clinician-in-the-loop evaluation, patient or clinician acceptability, alert burden, workflow integration, and explanation usability	Usability score, task-completion time, alert burden, override rate, clinician agreement, user trust, acceptability survey, EHR or telemedicine integration evidence	Assesses whether outputs can be used safely and meaningfully in real clinical workflows
Clinical-translation metrics	Safety, governance, and regulatory readiness	Error analysis, safety monitoring, model-version control, update policy, rollback process, audit trail, and regulatory planning	Error cases, adverse events or near misses, fail-safe behaviour, human approval for updates, model versioning, rollback log, monitoring thresholds, regulatory pathway	Provides cautious evidence for responsible translation without claiming routine clinical readiness

## 9 Conclusion

This review synthesized 55 peer-reviewed journal articles on neuro-fuzzy systems in IoMT and connected healthcare. The evidence shows clear promise for prediction, detection, monitoring, and early decision-support tasks. It also shows that the field remains dominated by static, retrospective, benchmark-driven, simulation-based, and prototype-level models. The article-level taxonomy links architecture, application domain, deployment environment, and evaluation practice, making the gap between algorithmic performance and practical readiness easier to trace. Most included articles report conventional predictive metrics, while few report latency, energy use, memory footprint, communication overhead, robustness, interoperability, clinician usability, safety, or prospective validation. The current evidence therefore does not establish routine clinical readiness or clinical effectiveness. Future work should combine transparent reporting with deployment-aware evaluation, interpretability assessment, auditability, prospective validation, safety assessment, and integration with electronic health record and telemedicine workflows.
