# Supplementary material for: Neuro-fuzzy systems in internet of medical things: a systematic review on applications, taxonomy, challenges and open issues
Source: Front Digit Health. 2026 Jul 27;8:1857965. doi: 10.3389/fdgth.2026.1857965 (PMC13454051; doi:10.3389/fdgth.2026.1857965)
Supplement: Supplementary file 1 [file Datasheet1.pdf]

Supplementary Material

1 SUPPLEMENTARY TABLES S1 AND S2

Supplementary Table S1 provides the article-level evidence base for the systematic review of neuro-fuzzy systems in Internet of Medical Things and connected-healthcare contexts. The supplementary workbook contains only the 55 peer-reviewed journal articles retained in the final systematic evidence base and supports traceability between individual articles and the aggregate synthesis reported in the manuscript. Reviews, conference papers, books, reports, standards, and other non-journal sources cited in the manuscript are background literature only and are not included in this workbook, the study-selection counts, the quality appraisal, or the quantitative synthesis.

Supplementary Table S2 provides the database-specific search audit used to strengthen reproducibility. It reports source-specific search modes, field restrictions, platform-adapted strings, filters, search dates, records retrieved, retained records, full-text assessment counts, final included counts, Google Scholar phrase-level handling, and duplicate/source-attribution notes.

The supplementary workbook is submitted as `NFS_IoMT_Supplementary_Tables_S1_S2.xlsm`. It contains the following sheets:

- **Supplementary Table S1**: article-level extraction matrix with citation, year, reference details, disease or application area, application category, data source or dataset, dataset type, neuro-fuzzy system type, static or evolving classification, architecture category, deployment environment, evaluation tool, reported metrics and results, latency reporting, energy reporting, memory or computational-resource reporting, deployment-sensitive evaluation, main limitation, and verification search URL.
- **Summary**: aggregate counts and percentages for architecture categories, application categories, static/evolving classification, latency reporting, energy reporting, memory or computational-resource reporting, source distribution, selection counts, quality-score distributions, deployment-fidelity distributions, primary deployment categories, medical-domain categories, and reviewer-driven consistency notes. All aggregate percentages use 55 as the denominator. Primary architecture, application-domain, medical-domain, and deployment percentages are mutually exclusive and sum to 100%, subject to rounding. Descriptive reporting fields such as latency, energy, memory, and performance metrics are not expected to sum to 100%.
- **Quality assessment**: article-level dataset adequacy, benchmarking rigor, deployment fidelity, aggregate quality score, NFS–IoMT evidence-quality category, scoring justification, quality-score distribution, deployment-fidelity distribution, and clinical-readiness caution. The low, moderate, and high labels refer to evidence quality for this review's NFS–IoMT synthesis and should not be read as general quality judgements on the original articles or on their contributions to the broader neuro-fuzzy systems field.
- **Supp Table S2 Search Audit**: complete database-specific reproducibility audit, including exact source-specific search strings, field restrictions or search modes, filters and limits, search dates, records retrieved, records entered after source-level filtering, title/abstract records retained, full texts assessed, final included journal articles, Google Scholar phrase-level handling, and source-specific notes.
- **Search concept blocks**: method, IoMT/connected-health, and task/application concept blocks used to construct and adapt the database searches.

- **S1 Search Audit**: source-level search summary corresponding to the main manuscript's source-audit table.
- **Selection audit summary**: study-selection counts corresponding to the structured flow diagram, including publisher records, screening counts, full-text assessment counts, final inclusion, and Google Scholar verification results.

The supplementary workbook also clarifies the interpretability framing used in the manuscript. Formal fuzzy-rule structure is treated as a basis for evaluation, not as automatic evidence of practical clinical interpretability. Future studies should report rule-base size, rule stability, clinician readability, explanation fidelity, and auditability, with additional version history and audit logs for evolving systems.

The workbook also documents how broader connected-health search terms were controlled during screening. These terms were used to improve retrieval sensitivity, but final inclusion required both a relevant neuro-fuzzy or fuzzy-inference method and a connected-health or IoMT-relevant healthcare context. General healthcare AI articles without a sensor-based, wearable, networked, remote-monitoring, or edge/fog/cloud health-monitoring component were excluded from the systematic evidence base.

2 AUTHOR-GUIDELINE CONSISTENCY CHECK

The revised project was checked against Frontiers article-type limits provided for review and systematic-review manuscripts. The abstract remains within the 350-word limit, the main manuscript text remains within the 12,000-word limit based on `texcount`, and the combined number of main-manuscript figures and tables remains within the expected display-item limit. Background-only comparison material and phrase-level Google Scholar detail are handled in the supplementary audit material rather than as additional main-manuscript display items.

The response package was also edited for consistency. Each response identifies the manuscript section or supplementary file revised for the corresponding reviewer issue, while avoiding repeated stock phrasing. The supplementary workbook has been kept as the central audit file for article-level extraction, quality assessment, search reproducibility, exclusion reasons, percentage checks, and reviewer-driven consistency notes.
